# Exploring coral microbiome assemblages in the South China Sea

**DOI:** 10.1038/s41598-018-20515-w

**Published:** 2018-02-05

**Authors:** Lin Cai, Ren-Mao Tian, Guowei Zhou, Haoya Tong, Yue Him Wong, Weipeng Zhang, Apple Pui Yi Chui, James Y. Xie, Jian-Wen Qiu, Put O. Ang, Sheng Liu, Hui Huang, Pei-Yuan Qian

**Affiliations:** 1 Shenzhen Research Institute and Division of Life Science, The Hong Kong University of Science and Technology, Hong Kong SAR, China; 2 Key Laboratory of Tropical Marine Bio-resources and Ecology, South China Sea Institute of Oceanology, Chinese Academy of Sciences, Guangzhou, China; 3 Marine Science Laboratory, Department of Biology, The Chinese University of Hong Kong, Hong Kong SAR, China; 4 Department of Biology, Hong Kong Baptist University, Hong Kong SAR, China

## Abstract

Coral reefs are significant ecosystems. The ecological success of coral reefs relies on not only coral-algal symbiosis but also coral-microbial partnership. However, microbiome assemblages in the South China Sea corals remain largely unexplored. Here, we compared the microbiome assemblages of reef-building corals *Galaxea* (*G. fascicularis*) and *Montipora* (*M. venosa*, *M. peltiformis*, *M. monasteriata*) collected from five different locations in the South China Sea using massively-parallel sequencing of 16S rRNA gene and multivariate analysis. The results indicated that microbiome assemblages for each coral species were unique regardless of location and were different from the corresponding seawater. Host type appeared to drive the coral microbiome assemblages rather than location and seawater. Network analysis was employed to explore coral microbiome co-occurrence patterns, which revealed 61 and 80 co-occurring microbial species assembling the *Galaxea* and *Montipora* microbiomes, respectively. Most of these co-occurring microbial species were commonly found in corals and were inferred to play potential roles in host nutrient metabolism; carbon, nitrogen, sulfur cycles; host detoxification; and climate change. These findings suggest that the co-occurring microbial species explored might be essential to maintain the critical coral-microbial partnership. The present study provides new insights into coral microbiome assemblages in the South China Sea.

## Introduction

Coral reef ecosystems are considered as the tropical rainforests of the sea, nurturing the highest biodiversity of marine life and providing vital ecosystem goods and services^1,2^. However, coral reefs around the world have suffered from declines and extinction risks largely due to bleaching events and emerging/reemerging diseases induced by climate change and anthropogenic disturbances^3,4^. The ecological success of coral reefs relies on coral-algal symbiosis, and recent studies using 16S rRNA gene amplicon pyrosequencing have revealed highly diverse and abundant microbes in individual coral colonies^5–9^. It is believed that some of these microbes can form partnerships with coral hosts and help them with possible access to those unavailable nutrients and metabolic pathways^10^. Compared with the well-understood coral-algal symbiosis, current understanding of coral-microbial partnership is rather limited. Although members of *Endozoicomonas*^6^ and *Prosthecochloris*^11^ have been shown to form potential symbioses with corals, no obligate coral-microbial symbiosis has been addressed to date. A recent advance in calcification for coral symbiotic algae demonstrated that free-living *Symbiodinium* in culture could form algal-microbial partnership, which facilitated the *Symbiodinium* calcification^12^. Taken these evidences, potentially complex coral-algal-microbial interactions in holobionts might facilitate the ecological success of coral reefs.

The coral mucus, tissue, and skeleton all contain large populations of associated microbes from three domains of life, including *Eukarya*, *Archaea*, and *Bacteria*, as well as many viruses^13,14^. The term coral microbiome is employed herein to collectively refer to the bacteria and archaea in coral holobionts. The characterization of coral core microbiomes reveals that two most abundant bacterial phylotypes affiliating to the genera *Propionibacterium* and *Ralstonia* are co-localized specifically with the host’s endosymbiotic algae which are likely to facilitate the success of coral-algal symbiosis^10^. Coral microbiomes are highly complex and dynamic, usually changing with environmental conditions, host types, and tempo-spatial gradients^5,15^. However, we assumed that there would be co-occurring microbial species assembling coral microbiomes which might be fundamental for coral holobionts to maintain the critical coral-microbial partnership. To explore these potential microbial species, co-occurrence network analysis was conducted in this study. Network-based approaches have been widely applied in microbial ecology studies^16^ such as soil^17^, coral^18^, human gut^19^, marine sediment^20^, stream biofilm^21^, activated sludge^22^, marine biofilm^23^, and other natural and man-made environments.

In recent years, there has been a growing interest in coral microbiome studies^24–28^, but very limited knowledge has been gained in understanding the microbiome assemblages for the South China Sea corals. We do not yet clearly understand how coral microbiomes are assembled in the South China Sea and potential co-occurring microbial species making up the coral microbiomes. The aims of this study were (i) to compare the microbiome assemblages using congeneric corals collected from different locations of the South China Sea, and (ii) to explore potential co-occurring microbial species through co-occurrence network analysis. We believe the present study facilitates our understanding of coral-microbial partnership in the South China Sea and serves as a useful reference for comparison with other regional coral microbiome assemblages.

## Results

### Microbiome assemblages and comparisons for *Galaxea* and *Montipora* at a high taxonomic level

**Figure 1:**
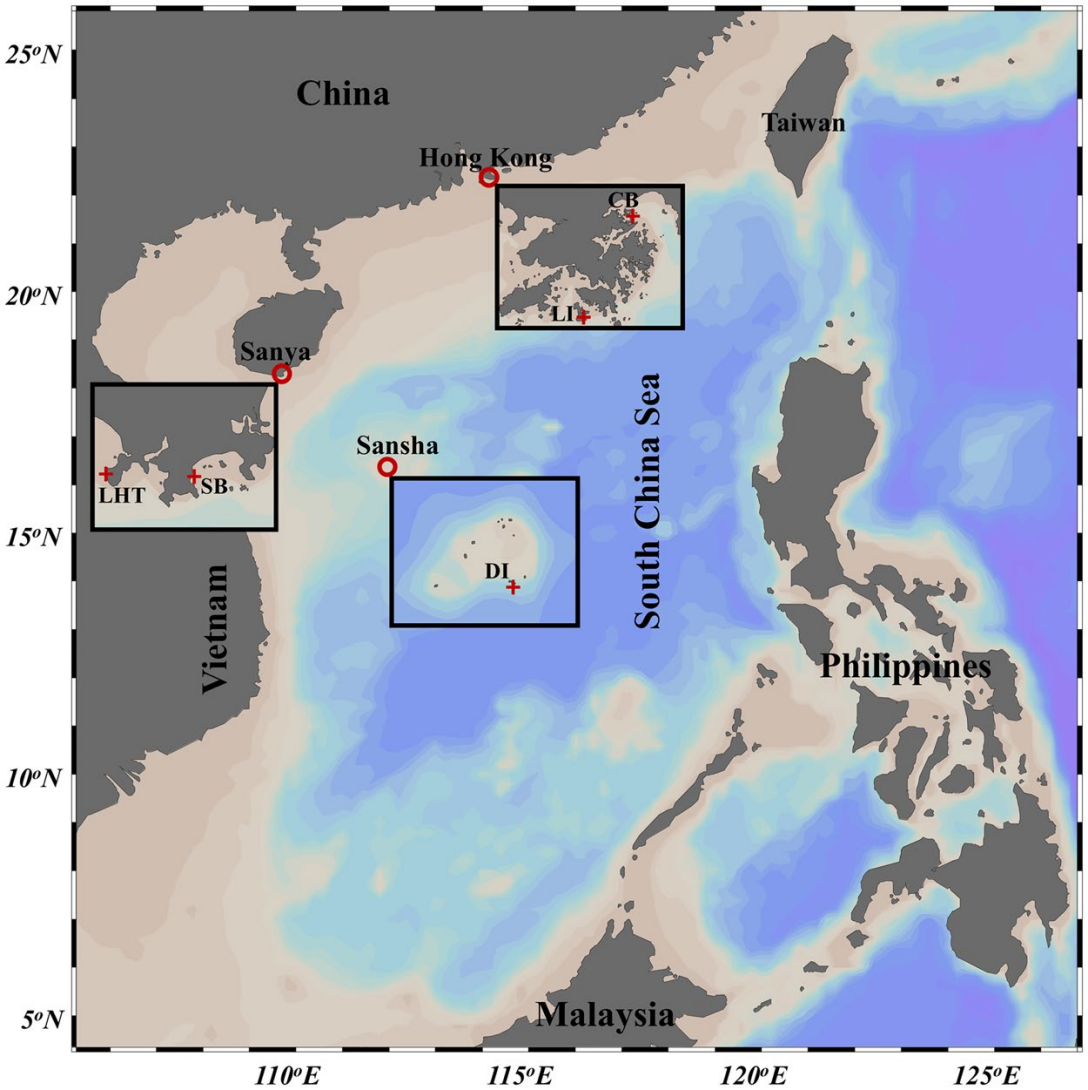
A geographic map of coral sampling locations in the South China Sea. The map showing dark gray, sandy, and blue indicates the land, shallow water, and deep water, respectively. The rectangles show the magnified areas of the red cycles. The red cycles and crosses indicate the sampling regions and locations, respectively. *Galaxea* and *Montipora* from three biogeographic regions in Hong Kong (Crescent Bay and Lamma Island, abbreviated as CB and LI), Sanya (Luhuitou and Sunny Bay, abbreviated as LHT and SB), and Sansha (Drummond Island, abbreviated as DI) were collected for this study. The map was plotted by Ocean Data View 4.7.2 (Schlitzer, R., Ocean Data View, odv.awi.de, 2017).

**Figure 2:**
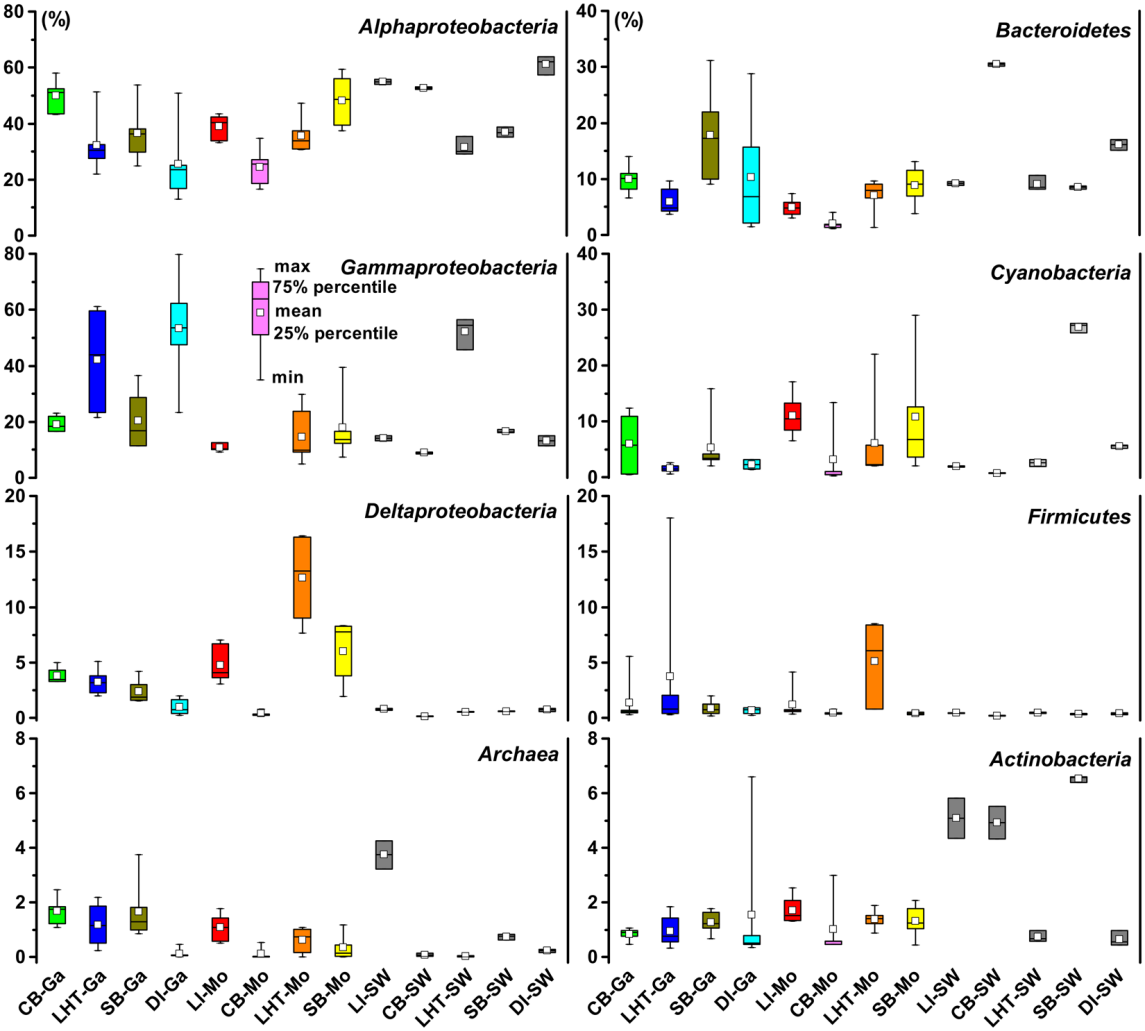
Coral microbiome assemblages at domain (only for *Archaea*), phylum, or class (only for *Proteobacteria*) level. Relative abundance for each taxon is plotted using a box chart statistically. The eight classified taxa *Alphaproteobacteria*, *Gammaproteobacteria*, *Deltaproteobacteria*, *Archaea*, *Bacteroidetes*, *Cyanobacteria*, *Firmicutes*, and *Actinobacteria* in Figure S1 were statistically analyzed. The detailed data are shown by stacked columns in Figure S1. CB, LI, LHT, SB, DI, Ga, Mo, and SW indicate Crescent Bay, Lamma Island, Luhuitou, Sunny Bay, Drummond Island, *Galaxea*, *Montipora*, and seawater, respectively.

After filtering unqualified sequences, a total of 587,425 clean 16S tags were obtained for the 59 coral and seawater samples collected from the five locations LI, CB, LHT, SB, and DI (Table 1 and Fig. 1). Detailed information for each sample, including the sample ID, number of clean 16S tags, number of observed species, and Shannon index, is shown in Table S1. The three technical replicates for coral colony LHT-Mo4 exhibited similar microbiome assemblages (Figure S1). The seawater technical replicates (collected in parallel) for the five sampling locations also displayed similar profiles (Figure S1). Both coral and seawater technical replicates demonstrated the sequencing data had a good reproducibility. As shown in Fig. 2, coral microbiome assemblages displayed quite different profiles among host type, location, and seawater at the domain, phylum, or class level. Individual colony microbiome assemblage also displayed varied patterns, even from the same species and location (Figure S1). The phylum *Proteobacteria* dominated all of the coral and seawater microbiome assemblages with a relative abundance greater than 50%, among which *Alphaproteobacteria* or *Gammaproteobacteria* accounted for the largest proportion (Fig. 2). This finding is consistent with the results of other studies on coral-associated microbes^7,8,18,29–32^. Relative abundance of *Deltaproteobacteria* was much higher in corals than that in seawater (Fig. 2). Compared with *Proteobacteria*, the phyla *Bacteroidetes*, *Cyanobacteria*, *Firmicutes*, and *Actinobacteria* were less abundant (Fig. 2), but they were essential members assembling the coral microbiomes. The domain *Archaea* was detected in both coral and seawater samples with a low relative abundance (Fig. 2), but most of them were ecologically significant ammonia-oxidizing archaea *Nitrosopumilus* spp.^33^.

**Table 1:** Information of sampling locations, coordinates, dates, species and relevant abbreviations used in this study. Abbreviations presented in parentheses were used as sample IDs of this study. A total of six healthy colonies were collected for each coral species at each sampling location. Seawater (SW) samples were also collected as reference.

Regions	Locations	Coordinates	Dates	*Galaxea* (Ga)	*Montipora* (Mo)
Hong Kong	Lamma Island (LI)	E114.135° N22.187°	19-Mar-14	absent	*M. venosa*
	Crescent Bay (CB)	E114.314° N22.531°	24-Mar-14	*G. fascicularis*	*M. peltiformis*
Sanya	Luhuitou (LHT)	E109.471° N18.212°	04-Apr-14	*G. fascicularis*	*M. monasteriata*
	Sunny Bay (SB)	E109.610° N18.199°	03-Apr-14	*G. fascicularis*	*M. monasteriata*
Sansha	Drummond Island (DI)	E111.778° N16.523°	19-Jun-14	*G. fascicularis*	absent

### Microbiome assemblages and comparisons for *Galaxea* and *Montipora* at a low taxonomic level

**Figure 3:**
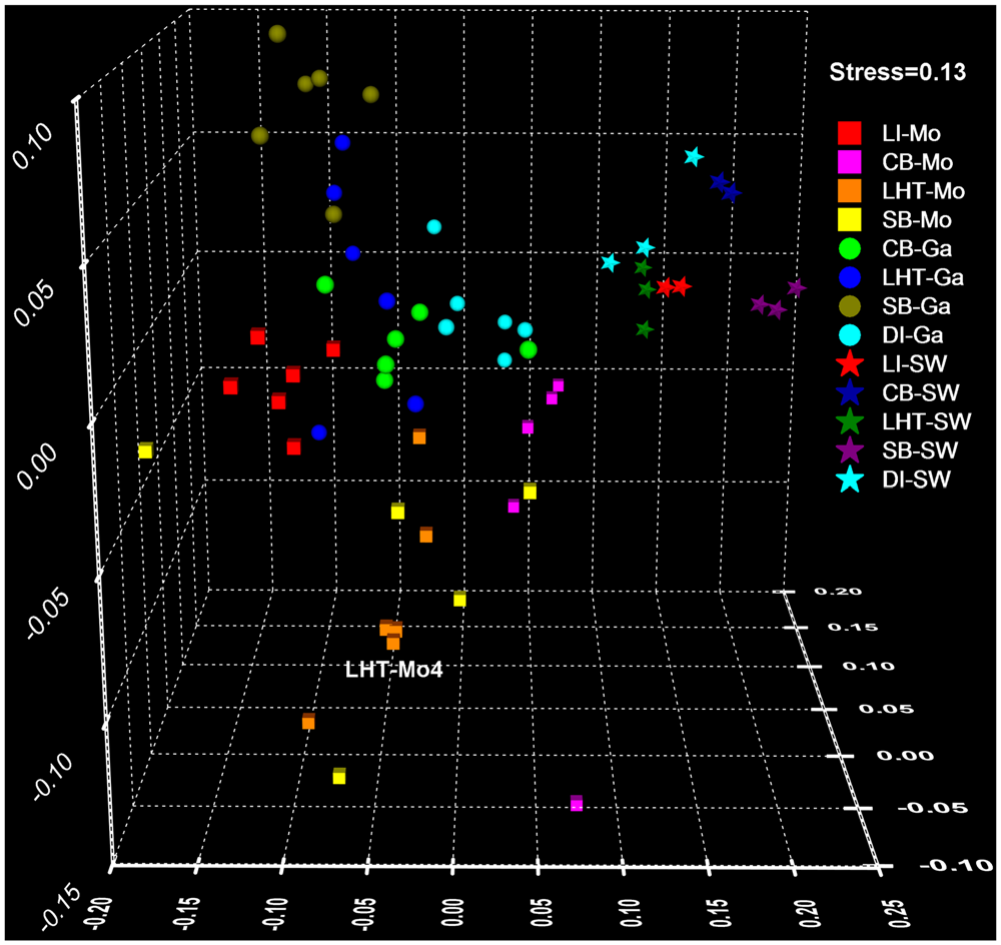
NMDS ordination for 97% OTU data of all samples using Bray-Curtis distance. The similarities of microbiome assemblages at species level among host type, sampling locations, and corresponding seawater were compared appropriately. CB, LI, LHT, SB, DI, Ga, Mo, and SW indicate Crescent Bay, Lamma Island, Luhuitou, Sunny Bay, Drummond Island, *Galaxea*, *Montipora*, and seawater, respectively.

The recovered 97% OTU data were used for statistical analyses to examine the differences (PERMANOVA) and similarities (NMDS) in coral microbiome assemblages between host type, location, and seawater. One-way PERMANOVA revealed that the *Galaxea* microbiome assemblages were significantly different among locations CB, LHT, SB, and DI (*P* < 0.01, *F* = 4.18), and the *Montipora* microbiome assemblages were also significantly different among locations LI, CB, LHT, and SB (*P* < 0.01, *F* = 6.23). To further explore the differences in coral microbiome assemblages between host type, location, and seawater comprehensively, thirteen groups (i.e. LI-Mo, LI-SW, CB-Ga, CB-Mo, CB-SW, LHT-Ga, LHT-Mo, LHT-SW, SB-Ga, SB-Mo, SB-SW, DI-Ga, and DI-SW) were generated for pairwise comparisons using one-way PERMANOVA. As shown in Table S2, coral microbiome assemblages for each host type from each location was significantly different from any other host type and the corresponding seawater (*P* < 0.05, 1.96 < *F* < 22.44). To explore the similarities among these thirteen groups, NMDS (stress = 0.13) was conducted and shown in Fig. 3. Colonies for each coral group (i.e. biological replicates) and the three technical replicates for LHT-Mo4 exhibited different distances, revealing that individual differences for the same host type and location can be either very high or very low. The five seawater groups were closely clustered and distinct from all coral groups, which is consistent with the previous findings^8,34^. The eight coral groups were loosely clustered together with four *Galaxea* groups and four *Montipora* groups distributed at the top and bottom respectively, indicating that host type exhibited a consistent distribution pattern. However, there was no consistent geographic pattern in distribution for both *Galaxea* and *Montipora* groups, suggesting that location contributed less in driving the coral microbiome assemblages. According to the NMDS result, host type but not location showed a consistent distribution pattern for the coral microbiome assemblages and the coral and seawater microbiomes were assembled dissimilarly. Therefore host type is suggested to play an even more important role than location and seawater in driving coral microbiome assemblages.

### Co-occurrence patterns for *Galaxea* and *Montipora* microbiomes

**Figure 4:**
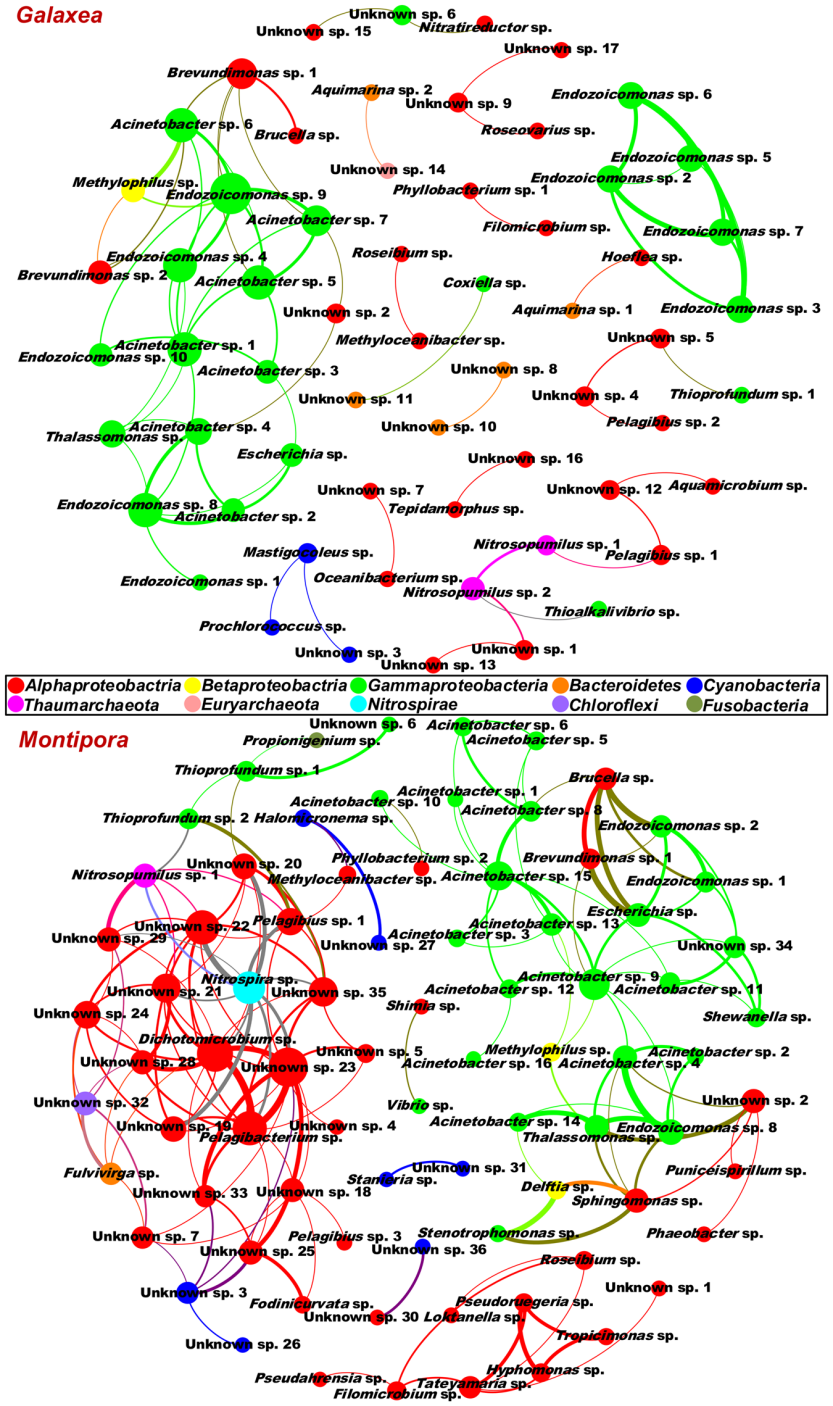
Co-occurrence patterns of microbial species assembling the *Galaxea* and *Montipora* microbiomes. Each connection indicates a strong and significant correlation, with the Spearman’s correlation coefficient higher than 0.6 and statistically significant (*P* < 0.01). Each node represents a microbial species, and its size is proportional to the node connectivity. Each edge represents a linkage between two co-occurring nodes, and its thickness is proportional to the Spearman’s correlation coefficients. All nodes are labeled with annotated species or unknown species, which are colored at the phylum level.

In total, 122 and 134 OTUs derived from the *Galaxea* and *Montipora* microbiomes were used for co-occurrence network analysis after removal of the poorly represented OTUs, respectively. We first tested the two OTU datasets and observed non-random co-occurrence patterns, which is consistent with other studies^17,19,20,22^. A study even demonstrated that non-random community assemblage might be a common feature of all life domains^35^. As shown in Fig. 4, the generated networks had 61 and 80 nodes (i.e., 97% OTUs or microbial species) and 73 and 172 edges (i.e., links between every two nodes) for the *Galaxea* and *Montipora* microbiomes, respectively. The network topological parameters for the *Galaxea* microbiomes included an average node connectivity of 2.39, an average network distance of 2.18 edges, a network diameter of 6 edges, an average clustering coefficient of 0.37, and a modularity index of 0.73. While the network topological parameters for the *Montipora* microbiomes contained an average node connectivity of 4.30, an average network distance of 2.76 edges, a network diameter of 6 edges, an average clustering coefficient of 0.48, and a modularity index of 0.62. These topological properties were used to describe the general structures of co-occurrence networks^17^. Both networks of the *Galaxea* and *Montipora* microbiomes had modular structures because each modularity index value was higher than 0.40^36^. Based on the modularity class, each network could be further divided into several sub-networks, for example, those closely interacted nodes on the left side of each network (Fig. 4).

There were 114 OTUs in the co-occurrence networks of the *Galaxea* and *Montipora* microbiomes (Fig. 4), among which 27 OTUs were shared between the two corals (Figures S2 and S3). These OTUs were taxonomically annotated by BLAST online search against the GenBank 16S rRNA gene database using 94% similarity cutoff (Table S3). OTUs that exhibited less than 94% similarity or any ambiguous match to a specific genus were annotated as unknown species. There were a total of 78 annotated species and 36 unknown species (Figs 4 and S2), most of which were affiliated to *Alphaproteobacteria* and *Gammaproteobacteria*. To achieve a basic understanding of the unknown species, phylogenetic analysis was conducted using their closest relatives (Figure S4), and the result showed that 27 of the 36 unknown species were phylogenetically close to the previously reported coral-associated microbes, and the others were phylogenetically related to marine bacteria (Figure S4 and Table S3). Among the 78 annotated species, 64 were reported as coral-associated microbes, and the remainders were also known as marine bacteria (Table S3). Because the 114 OTUs derived from the co-occurrence networks were demonstrated to have statistically significant interactions, most of which were identified as coral-associated microbes in this and previous studies (see Table S3 for details). These OTUs are believed to serve as necessary co-occurring microbial species assembling the coral microbiomes.

### Profiles of co-occurring microbial species of the *Galaxea* and *Montipora* microbiomes

**Figure 5:**
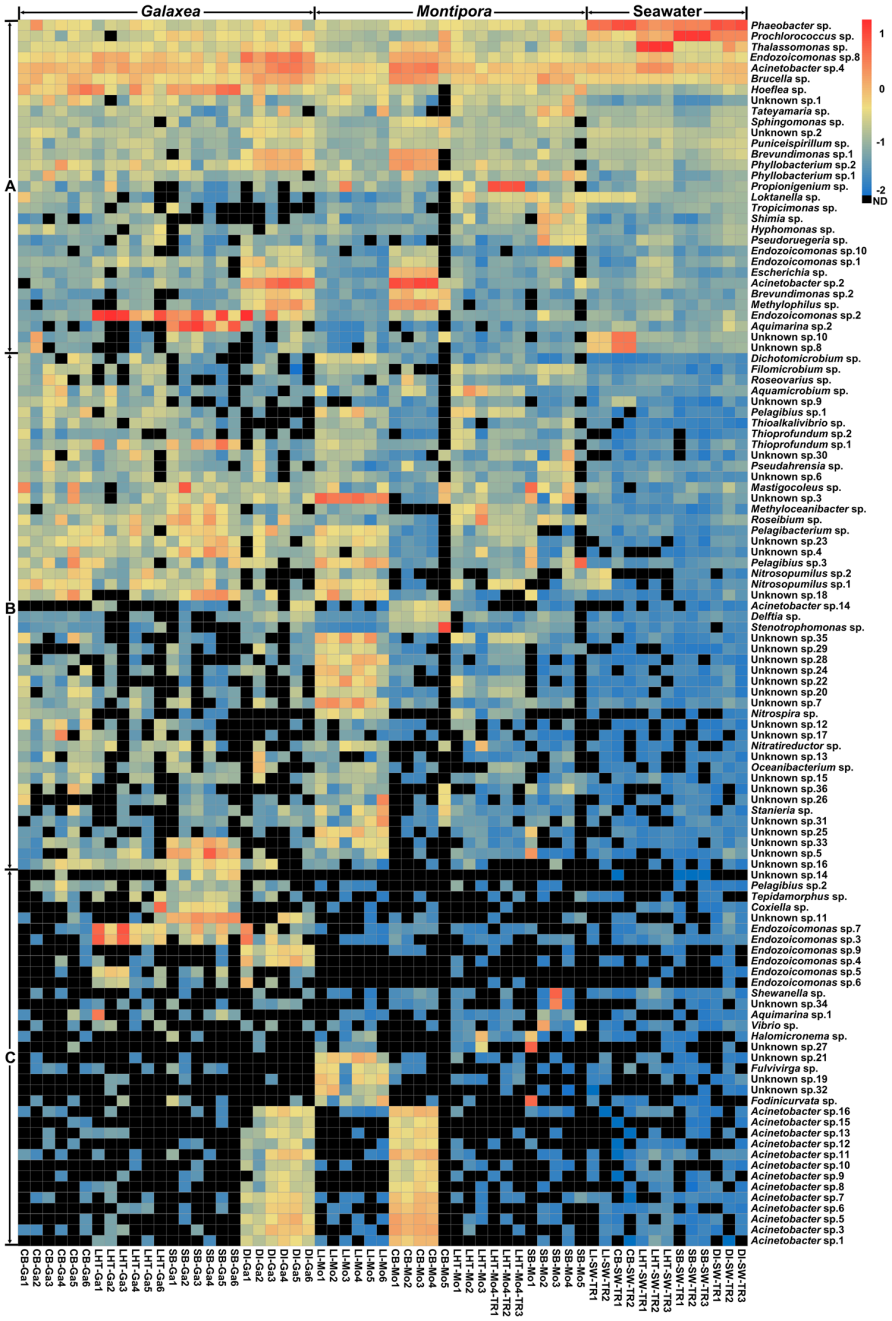
Profiles of co-occurring microbial species for the *Galaxea* and *Montipora* microbiomes. Relative abundance was log_10_-transformed for plotting. The scale bars ND, −2, −1, 0, and 1 indicate relative abundance of 0, 0.01%, 0.1%, 1%, and 10%, respectively. The bottom panel represents the sample IDs. The right panel shows the IDs of the co-occurring microbial species. CB, LI, LHT, SB, DI, Ga, Mo, SW, and TR indicate Crescent Bay, Lamma Island, Luhuitou, Sunny Bay, Drummond Island, *Galaxea*, *Montipora*, seawater, and technical replicate, respectively.

As shown in Fig. 4, the 61 co-occurring microbial species making up the *Galaxea* microbiomes were assigned to *Alphaproteobacteria* (26), *Betaproteobacteria* (1), *Gammaproteobacteria* (23), *Bacteroidetes* (5), *Cyanobacteria* (3), *Thaumarchaeota* (2), and *Euryarchaeota* (1). While the 80 co-occurring microbial species assembling the *Montipora* microbiomes were classified into *Alphaproteobacteria* (39), *Betaproteobacteria* (2), *Gammaproteobacteria* (27), *Bacteroidetes* (1), *Cyanobacteria* (7), *Thaumarchaeota* (1), *Nitrospirae* (1), *Chloroflexi* (1), and *Fusabacteria* (1). The relative abundance of the 114 co-occurring microbial species and the corresponding seawater samples is shown using a heat map (Fig. 5). The 31 species in Fig. 5A showed similar profiles between coral and seawater samples, revealing that they might multiply well in both corals and seawater. While the 48 species in Fig. 5B exhibited higher relative abundance in corals than that in seawater, suggesting that they might benefit from coral hosts and proliferated more efficiently in corals than in seawater. The 35 species in Fig. 5C displayed unusual profiles between corals from different locations, indicating that some of them might be enriched in corals from certain location. Most of the 114 co-occurring microbial species are known as heterotrophs, which might utilize holobiont organic wastes for growth. The remainders are autotrophs, including photoautotrophs like cyanobacteria and chemoautotrophs such as ammonia-oxidizing archaea *Nitrosopumilus* and sulfur-oxidizing bacteria *Thioalkalivibrio* and *Thioprofundum*. Cluster analysis for the co-occurring microbial species explored were performed to reveal the similarities between corals from different locations and between corals and seawater (Figure S5). Corals from the same location clustered preferentially with individual colony differences and they were relatively distant to the seawater, which is similar to the NMDS result.

There were 27 shared co-occurring microbial species assembling the *Galaxea* and *Montipora* microbiomes (Figures S2 and S3). As shown in Figs 4 and S3, they belonged to *Alphaproteobacteria* (11), *Betaproteobacteria* (1), *Gammaproteobacteria* (13), *Cyanobacteria* (1), and *Thaumarchaeota* (1). These shared co-occurring microbial species included 7 unknown spp., 6 *Acinetobacter* spp., 3 *Endozoicomonas* spp., and a species from each of the following 11 genera: *Brevundimonas*, *Brucella*, *Escherichia*, *Filomicrobium*, *Methyloceanibacter*, *Methylophilus*, *Nitrosopumilus*, *Pelagibius*, *Roseibium*, *Thalassomonas*, and *Thioprofundum*. These shared co-occurring microbial species assembling the *Galaxea* and *Montipora* microbiomes might be prevalent in other corals from the South China Sea.

## Discussion

In recent years, the concept of “core microbiome” has been introduced in coral microbial ecology studies^10,26,37^, while the counterpart “stable microbiome” has also been used in related studies^26,37,38^. According to the statistical methods used for data analysis, coral-associated microbes can be assigned into “core/stable microbiome” and “transient/sporadic microbiome”^37^. The “core microbiome” is described as the stable and consistent components across complex microbiome assemblages from similar habitats which can be determined with one of the five described variations, that is, a core based on shared presence, shared abundance, shared composition, phylogenetic information, or interaction^37,39^. Co-occurring microbial species explored here through co-occurrence network analysis are exactly equal to the “core microbiome” based on interaction, as illustrated in related studies^37,39^. In the present study, we collected coral samples with a large set of biological replicates from the five locations in the South China Sea, allowing us to compare the complex microbiome assemblages comprehensively and to explore co-occurrence patterns through network analysis. We further discussed the effects of host type and location in coral microbiome assemblages (the first section below) and potential roles of co-occurring microbial species explored including those functional microbes (the second section below) and those mostly found microbes (the third section below).

### Effects of host type and location in coral microbiome assemblages

Corals harbor highly diverse microbes that might be assembled similarly or dissimilarly among host types, locations and other potential determinants. The study on Caribbean corals shows that different hosts harbor distinct host-specific microbes and that specificity varies by host type and location^15^. The study on Red Sea corals reveals that the microbiome assemblages vary largely with location and are shaped by host type^5^. Certain microbes are reported to form species-specific associations with corals^40^, suggesting the roles of host played in driving coral microbiome assemblages. The study on corals from the Great Barrier Reef shows that certain microbes are associated with corals specifically and that microbiome assemblages differ with the location but not the host type^41^. These findings support the present study that host type rather than location drives the coral microbiome assemblages, as shown in Fig. 3. There is a limitation that three species from the genus *Montipora* were collected for the present study because the shared species were not found in the selected locations except for *Galaxea fascicularis*. The study using three species of the genus *Acropora* found that they harbored conserved microbes and suggested that closely related corals of the same genus were assembled with similar microbes^41^. So it can be assumed that if one of the three studied *Montipora* species were shared and used for the present study, the general distribution pattern of microbiome assemblages for this species from different locations might be less likely affected in the NMDS ordination. The distribution pattern of the three *Montipora* species shown in Fig. 3 further supports that host type rather than location drives the coral microbiome assemblages because LHT-Mo and SB-Mo from the same host species *Montipora monasteriata* were clustered closely and were relatively distinct from LI-Mo and CB-Mo (from *Montipora venosa* and *Montipora peltiformis* respectively).

### Potential roles of certain functional co-occurring microbial species

**Figure 6:**
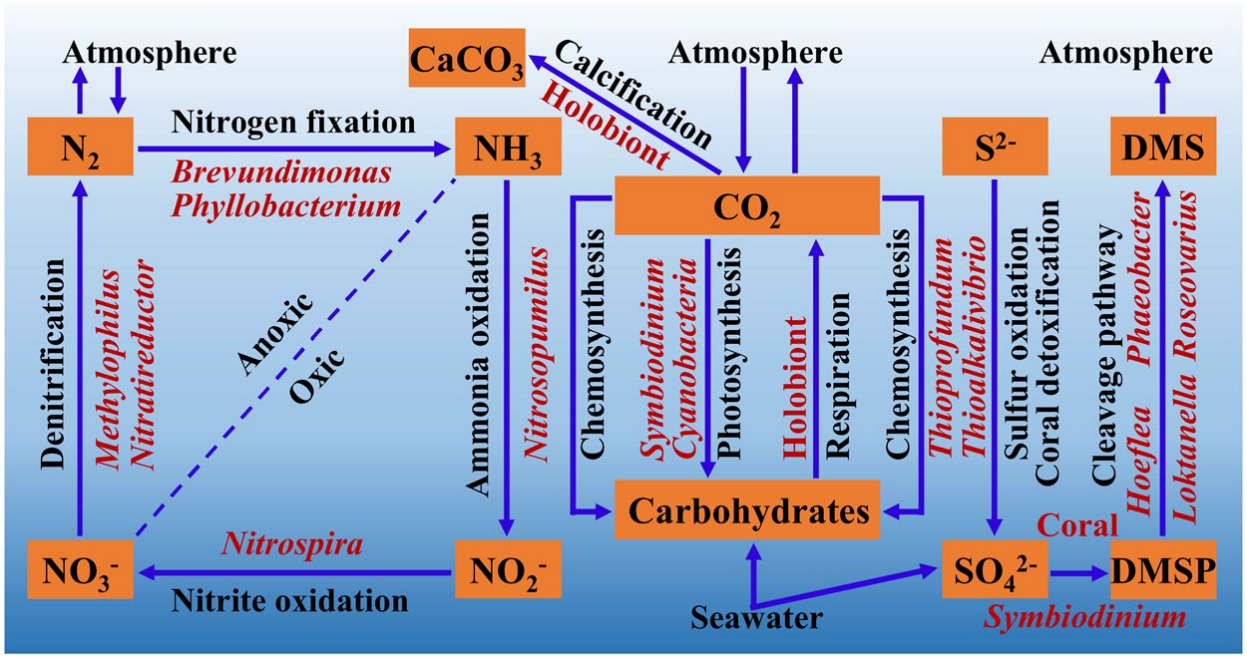
A schematic representation illustrating potential roles of certain co-occurring microbial species in corals. Potential roles might be played in host nutrient metabolism; carbon, nitrogen, sulfur cycles; host detoxification; and climate change. Those co-occurring microbial species shown in red were mapped onto the well-known ecological processes.

To achieve a better understanding of co-occurring microbial species, potential ecological roles and associations with coral hosts were further explored. As shown in Fig. 6, the listed co-occurring microbial species might be involved in several important biological and ecological processes: nutrient metabolism; carbon, nitrogen, and sulfur cycles; host detoxification; and climate change. Potential nitrogen-fixing bacteria such as *Brevundimonas* spp. and *Phyllobacterium* spp. might fix atmospheric nitrogen gas to ammonia. Potential ammonia-oxidizing archaea like *Nitrosopumilus* spp. might oxidize the holobiont ammonia to nitrite, and possible nitrite-oxidizing bacteria like *Nitrospira* sp. might oxidize the holobiont nitrite to nitrate. Finally, potential denitrifying bacteria such as *Methylophilus* sp. and *Nitratireductor* sp. might convert the holobiont nitrate to nitrogen gas. These co-occurring microbial species might thus be involved in the complete nitrogen cycle and changes in their population and activity might affect certain process of the nitrogen metabolism in coral holobionts^42^. It is suggested that they might serve as nitrogen regulators to keep the balance of holobiont nitrogen through providing sufficient bioavailable nitrogen and removing unneeded nitrogen. Carbon dioxide might be fixed into carbohydrates by *Symbiodinium* and *Cyanobacteria* through photosynthesis and by the co-occurring microbial species explored such as *Nitrosopumilus* spp., *Thioalkalivibrio* sp., and *Thioprofundum* spp. through chemosynthesis. Like *Symbiodinium*, the synthesized carbohydrates by the co-occurring microbial species might also serve as the host nutrients or food sources. The study has demonstrated that free-living *Symbiodinium* can calcify with the aid of microbial partners^12^, implying that potential co-occurring microbial species explored might also facilitate coral calcification directly or indirectly. Hydrogen sulfide is toxic to a wide range of eukaryotic organisms, including marine invertebrates like coral and sponge, by inhibiting cytochrome c oxidase and a type of catalase^43,44^. In addition, hydrogen sulfide generated by sulfate-reducing bacteria might lead to the initiation of coral black band disease, which has been consistently found in lab induction and field observation^45,46^. Co-occurring microbial species *Thioalkalivibrio* sp. and *Thioprofundum* spp., serving as potential sulfur-oxidizing bacteria, might oxidize holobiont accumulated hydrogen sulfide to sulfate, thus contributing to coral health through detoxification of reduced sulfur compounds. Both DMSP (dimethylsulfoniopropionate) and DMS (dimethylsulfide) are important compounds in the global sulfur cycle as they are closely related to cloud formation and global climate change^47^. Both reef-building corals and free-living *Symbiodinium* have been documented to be high producers of DMSP^48,49^. The generated DMSP might be degraded into climate-active gas DMS via the bacterial cleavage pathway by co-occurring microbial species explored such as *Hoeflea* sp., *Loktanella* sp., *Phaeobacter* sp., *Roseovarius* sp., *Shewanella* sp., and *Vibrio* sp.^47^. In summary, co-occurring microbial species explored might play potential roles in host nutrient metabolism; carbon, nitrogen, sulfur cycles; host detoxification; and climate change and might be essential to maintain the critical coral-microbial partnership. However, the real functions need to be tested in future studies. For example, microbial genome recovery using culture-independent methods such as genome binning and single-cell genomics is promising to validate the specific functions on a genome scale.

### Potential roles of the mostly found co-occurring microbial species *Acinetobacter* spp. and *Endozoicomonas* spp

Among the 114 co-occurring microbial species assembling the *Galaxea* and *Montipora* microbiomes, 16 *Acinetobacter* spp. and 10 *Endozoicomonas* spp. were found, of which 6 *Acinetobacter* spp. and 3 *Endozoicomonas* spp. were shared between the two corals. This finding corroborates the high occurrence of *Acinetobacter* spp. and *Endozoicomonas* spp. in coral microbiomes found in different coral species and regions (Table S3). However, the roles of *Endozoicomonas* spp. and *Acinetobacter* spp. are poorly understood to date. One study demonstrated that *Acinetobacter* spp. were dominant in the microbiomes of bleached corals but were not detected in healthy corals^50^. *Acinetobacter* spp. are not likely to serve as bleaching causing agents because they can also be detected in high abundance in healthy corals, as observed in the present and the other studies^7,8,15,34^. It is important to note, however, that *Acinetobacter baumannii* is a highly troublesome human pathogen worldwide due to its resistance to antibiotics^51^. This implies that potential *Acinetobacter* sp. might be a threat to coral hosts. An early study demonstrated that *Acinetobacter guillouiae* strain 20B is a DMS oxidizer^52^, suggesting that *Acinetobacter* spp. might participate in holobiont DMS metabolism. However, more and more recent studies have demonstrated that *Acinetobacter* spp. have the denitrification function, for example, *Acinetobacter baumannii* H1^53^, *Acinetobacter johnonii* DBP-3^54^, *Acinetobacter* sp. HA2^55^, *Acinetobacter* sp. YZS-X1–1^56^, and *Acinetobacter* sp. SZ28^57^.

Besides the present study, *Endozoicomonas* spp. have been reported to be dominant in the microbiome assemblages of multiple coral species such as *Porites astreoides*^15^, *Stylophora pistillata*^6^, *Acropora millepora*^31^, *Seriatopora hystrix*^32^, and *Coelastrea aspera*^58^. Several *Endozoicomonas* species have been isolated from different marine invertebrates including a sponge^59^, a sea anemone^60^, a sea slug^61^, a comb pen shell^62^, and an octocoral^63^, and a stony coral^64^. *Endozoicomonas* spp. might play certain roles in their hosts because they are associated with a broad range of marine invertebrates. The roles of *Endozoicomonas* spp. in corals have been inferred in several studies^31,32,65^. Here PICRUSt prediction using 16S sequences assigned to *Endozoicomonas* spp. revealed an unreported function that they have a complete denitrification pathway including genes *napAB*, *nirK*, *norBC*, and *nosZ* responsible for converting nitrate to nitrite, nitrite to nitric oxide, nitric oxide to nitrous oxide, and nitrous oxide to nitrogen, respectively. However, further experiments are needed to elucidate their real roles in corals. In addition, *Endozoicomonas* spp. might be developed as coral health indicators because they are distributed extensively in corals and other marine invertebrates and no negative effects on corals have been reported to date.

## Conclusions

In the present study, we examined the coral microbiome assemblages associated with *Galaxea* and *Montipora* collected from five locations in three biogeographic regions of the South China Sea. The highly dominant *Proteobacteria* and less abundant *Bacteroidetes*, *Cyanobacteria*, *Firmicutes*, and *Actinobacteria* are the main phyla in structuring the coral microbiomes. OTU-based multivariate statistical analysis demonstrated that each host species from each location was significantly different from any other host species and the corresponding seawater in the coral microbiome assemblages. Host type appeared to drive the coral microbiome assemblages rather than location and seawater. The network analysis explored a set of co-occurring microbial species assembling the coral microbiomes, which might play potential roles in host nutrient metabolism; carbon, nitrogen, sulfur cycles; host detoxification; and climate change. The findings of this study form a baseline for assessing coral microbiome development in the South China Sea, and for microbiome assemblage comparison across regions. The present study extends our knowledge of coral microbiome assemblages in the South China Sea and facilitates our understanding of coral-microbial partnership.

## Methods

### Sampling locations and corals

Three cities Hong Kong, Sanya, and Sansha, representing three different biogeographic regions of the South China Sea for hard coral growth, were selected for the present study (Fig. 1). Hong Kong’s climate is subtropical with seasonal sea surface temperature (SST) ranging from 14 °C in winter to 31 °C in summer^66^, which is a marginal environment for the development of coral communities. Hard corals of Hong Kong grow very slowly, and are unable to form real reefs. In contrast to Hong Kong, both Sanya and Sansha are tropical climates with a mean SST of 22.5 °C and 23.8 °C in winter and 30.0 °C and 29.8 °C in summer, respectively^67^. Sansha has a tropical climate for coral development, while Sanya is located in a transitional zone between tropical and subtropical climates. As shown in Fig. 1, two sampling locations were selected for Hong Kong (Lamma Island and Crescent Bay, abbreviated as LI and CB) and Sanya (Luhuitou and Sunny Bay, abbreviated as LHT and SB), but only one for Sansha (Drummond Island, abbreviated as DI) because of its simple and original environment. Lamma Island, close to the Pearl River, is a tough estuarine environment for coral survival. Crescent Bay, located in the Mirs Bay region, is a normal oceanic environment for coral growth. The coral cover of Luhuitou has significantly decreased for decades due to the frequent human activities and pollution, while Sunny Bay corals are well protected by the Sanya Coral Reef National Nature Reserve^68^. *Galaxea* and *Montipora* were used as the target corals of the present study because they are commonly distributed in Indo-Pacific region and they are also found in most of the selected sampling locations (Table 1). These conspecific and congeneric corals were thus collected from the three selected biogeographic regions, and in two of these regions with two locations of contrasting environmental conditions, which were used to explore the coral microbiome assemblages in the South China Sea, potential co-occurring microbial species and their roles in coral holobionts.

### Coral collection

Information for each sampling location including location names, coordinates, sampling dates, coral species, and abbreviations is shown in Table 1. At each sampling, a small piece of coral tissue (~1 cm × 1 cm) from an apparently healthy colony was collected using a hammer & chisel and packaged into a tagged sterile bag. A total of six colonies representing biological replicates were collected for each coral species at each sampling location. After sampling, the coral specimens were immediately washed using sterile seawater to remove loosely attached microbes and fixed in 70% ethanol. All of the fixed coral specimens were kept with dry ice in an icebox. Seawater surrounding the sampled coral colonies was also collected in parallel for comparison with the corresponding coral samples. The microbes within 1 L seawater were concentrated by filtering the water through a 0.22-μm polycarbonate membrane using Pall Gelman 6-Place Aluminum Vacuum Filter Funnel Manifold #15403 and fixed with 50% ethanol. After fixation, all of the samples were stored at −30 °C until used for DNA extraction.

### DNA extraction, PCR, and high-throughput sequencing

A small fraction (~0.5 cm × 0.5 cm) of each coral specimen fixed in ethanol was first rinsed with 1× PBSE (137 mM NaCl, 2.7 mM KCl, 4.3 mM Na_2_HPO_4_·7H_2_O, 1.4 mM KH_2_PO_4_ and 10 mM EDTA) and ground in 1× PBSE using a mortar & pestle. The resulting coral slurry was centrifuged at 12,000 g for 5 min to collect all pellets for total DNA extraction using the FastDNA^®^ Spin Kit for Soil (MP Biomedicals, France). The DNA extracts were used as PCR templates after quality and purity determination through agarose gel electrophoresis and OD_260_/OD_280_ ratio measurement. To amplify and sequence the hypervariable V3 and V4 regions of the 16S rRNA gene, a universal primer set, 341 F (5′-CCTAYGGGRBGCASCAG-3′) and 802 R (5′-TACNVGGGTATCTAATCC-3′), was used for PCR amplification^69,70^. Unique barcodes of six nucleotides were modified at the 5′ terminus of the forward primer 341 F to allow multiplexed sequencing^71^. PCR was conducted using the following program: initial denaturation at 94 °C for 5 min, followed by 30 cycles at 94 °C for 0.5 min, 50 °C for 0.5 min, 72 °C for 1 min, and a final extension at 72 °C for 5 min. Each PCR reaction was pooled with ~50 ng DNA template, 200 nM of each primer, 25 μL 2× Premix Ex Taq solution (TaKaRa, China), and ddH_2_O up to 50 μL. To minimize potential PCR bias, three independent reactions were made for each sample and the products were pooled for purification using the PureLink^®^ PCR Purification Kit (Invitrogen, USA). The purified PCR products were quantified using a Thermo NanoDrop 2000 UV-Vis Spectrophotometer and pooled at equivalent concentrations for subsequent multiplexed sequencing of 16S amplicons. The pooled DNA sample was sent to Novogene (Beijing, China) for sequencing on an Illumina MiSeq sequencer with a paired-end (PE) mode and a read length of 300 bp. Because the length of the 16S V3V4 fragment is approximately 450 bp, paired-end 300 bp sequencing is sufficient to form overlaps for full-length amplicon assembly^72,73^. The sequencing datasets have been deposited in the NCBI Sequence Read Archive under accession number SRP066229. Totally, there were 59 samples sequenced for the five locations LI, CB, LHT, SB, and DI (Table 1 and Fig. 1): 6 samples of *Galaxea* for each location of CB, LHT, SB, and DI; 4–6 samples of *Montipora* for locations of LI (6), CB (5), LHT (4), and SB (5); 2 additional *Montipora* technical replicates for the location of LHT, and 2–3 technical replicates of seawater samples for locations of LI (2), CB (2), LHT (3), SB (3), and DI (3). There were four *Montipora* samples failed in the DNA extraction or PCR amplification, that is, each one from the locations of CB and SB and two from the location of LHT.

### Data trimming and bioinformatics analysis

The raw data were first trimmed to remove sequencing adaptors, short reads (<300 bp), and low quality reads (average quality score <20). To obtain the full-length of 16S V3V4 sequences, the PEAR (paired-end read merger) tool^74^ was employed to merge the overlapping PE reads into 16S tags using default parameters. The QIIME (quantitative insights into microbial ecology) platform^75^ was used to demultiplex 16S tags into individual samples identified by unique barcodes. Potential PCR chimeras were detected and removed using the ChimeraSlayer^76^ command in the QIIME. After strict data trimming, the clean 16S tags were recovered and normalized for the downstream analysis. OTUs (operational taxonomic units) at 97% similarity (equal to the species level) were clustered and annotated using the QIIME pipeline under default settings. Relative abundance data for community structures were automatically generated from phylum to genus. A 97% OTU matrix was recovered from the generated OTU table of biom file format, and the data were used for species-level permutational multivariate analysis of variance (PERMANOVA), non-metric multidimensional scaling (NMDS), and network analysis. Those poorly represented OTUs (mean relative abundance lower than 0.1%) were removed before the network analysis as previously described^17^. Briefly, microbial species-level co-occurrence networks were conducted in the R^77^ environment using the vegan^78^, igraph^79^, and Hmisc^80^ packages. Positive co-occurrence events were considered if the OTUs demonstrated the Spearman’s correlation coefficient higher than 0.6 and statistically significant (*P* < 0.01)^17,21^. The constructed networks were further optimized by the interactive visualization platform Gephi^81^. The R software was also employed for the heat map plotting to visualize profiles of the co-occurring microbial species in each sample. MEGA 6.06^82^ was used to construct the phylogenetic tree of 16S rRNA gene. PAST 3.10^83^ was used for PERMANOVA, NMDS and cluster analysis based on the Bray-Curtis distance. OriginPro 9.0 was employed for interactive scientific graphing. PICRUSt (Phylogenetic Investigation of Communities by Reconstruction of Unobserved States) prediction was conducted following the established pipeline^84^.

## Electronic supplementary material

Supplementary Information
