# Supplementary material for: Exploring coral microbiome assemblages in the South China Sea
Source: Sci Rep. 2018 Feb 5;8:2428. doi: 10.1038/s41598-018-20515-w (PMC5799258; doi:10.1038/s41598-018-20515-w)
Supplement: Supplementary file 1 — Supplementary Information [file 41598_2018_20515_MOESM1_ESM.pdf]

Supplementary Information

Exploring coral microbiome assemblages in the South China Sea

Lin Cai[1], Ren-Mao Tian[1], Guowei Zhou[1,2], Haoya Tong[1], Yue Him Wong[1], Weipeng Zhang[1], Apple Pui Yi Chui[3], James Y. Xie[4], Jian-Wen Qiu[4], Put O. Ang[3], Sheng Liu[2], Hui Huang[2,*], Pei-Yuan Qian[1,*]

[1] Shenzhen Research Institute and Division of Life Science, The Hong Kong University of Science and Technology, Hong Kong SAR, China

[2] Key Laboratory of Tropical Marine Bio-resources and Ecology, South China Sea Institute of Oceanology, Chinese Academy of Sciences, Guangzhou, China

[3] Marine Science Laboratory, Department of Biology, The Chinese University of Hong Kong, Hong Kong SAR, China

[4] Department of Biology, Hong Kong Baptist University, Hong Kong SAR, China

Running title: Coral microbiome assemblages in the South China Sea

***Corresponding author:**

Hui Huang, PhD, Senior Scientist
South China Sea Institute of Oceanology, Chinese Academy of Sciences, 164 West Xingang Road, Guangzhou, China
Phone & Fax: +862-8446-0294 E-mail: huanghui@scsio.ac.cn

Pei-Yuan Qian, PhD, Chair Professor
Division of Life Science, The Hong Kong University of Science and Technology, Clear Water Bay, Hong Kong SAR, China
Tel: +852-2358-7331 Fax: +852-2358-1559 E-mail: boqianpy@ust.hk

List of contents:

Figure S1 Relative abundance at domain (only for *Archaea*), phylum, or class (only for *Proteobacteria*) level.

Figure S2 Summary for co-occurring microbial species of *Galaxea* and *Montipora* microbiomes.

Figure S3 Co-occurrence patterns of microbial species assembling the *Galaxea* and *Montipora* microbiomes.

Figure S4 Phylogenetic analysis for 36 unknown co-occurring microbial species derived from *Galaxea* and *Montipra* microbiomes.

Figure S5 Cluster analysis for co-occurring microbial species derived from *Galaxea* microbiome (A) and *Montipra* microbiome (B).

Table S1 Identifications, number of clean 16S tags, number of species, and Shannon index summarized for each sample.

Table S2 *P* and *F* value of one-way PERMANOVA for pairwise comparisons between species, locations, and seawater.

Table S3 Information of 114 co-occurring microbial species involved in coral microbiome assemblages.

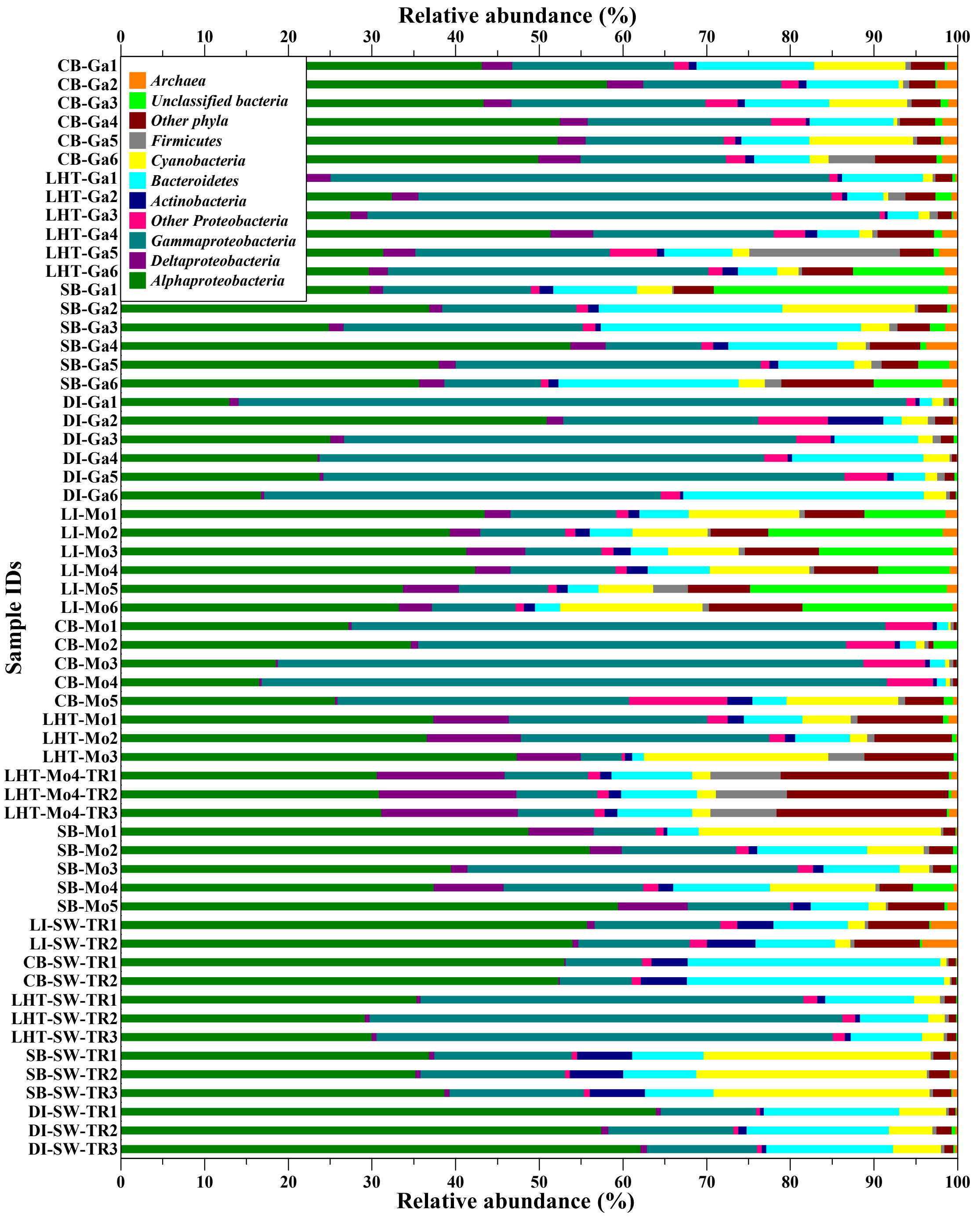

Figure S1 Relative abundance at domain (only for *Archaea*), phylum, or class (only for *Proteobacteria*) level. Bacteria that could not be classified at the phylum level were labeled "Unclassified bacteria". Phyla with a very low abundance were denoted "Other phyla".

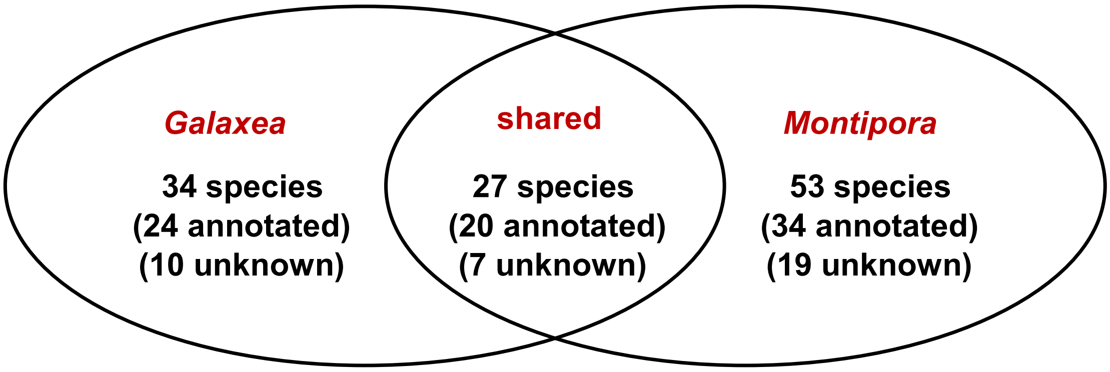

Figure S2 Summary for the co-occurring species of *Galaxea* and *Montipora* microbiomes.

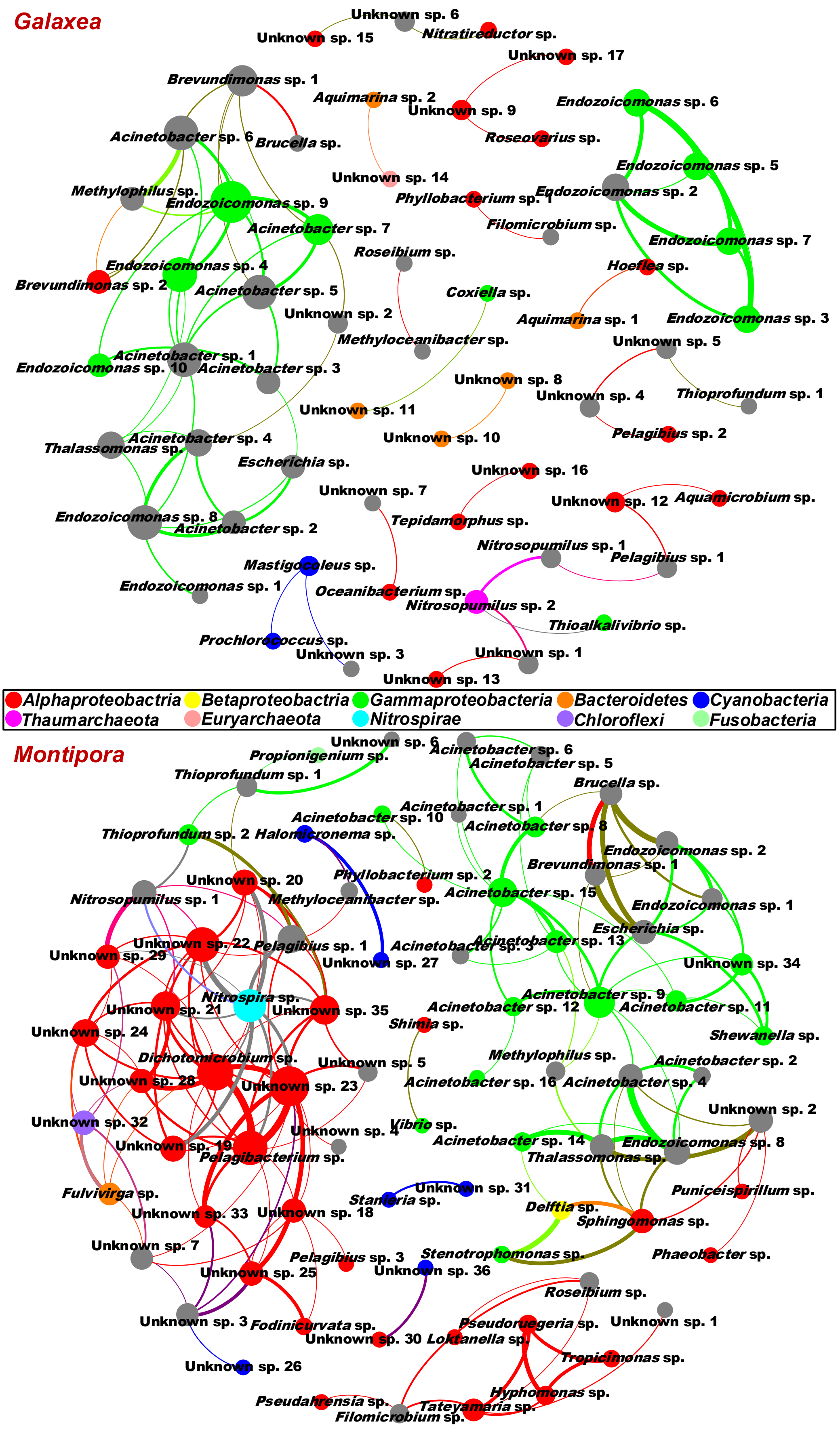

Figure S3 Co-occurrence patterns of microbial species assembling the *Galaxea* and *Montipora*

microbiomes. Each connection indicates a strong and significant correlation, with the Spearman's correlation coefficient higher than 0.6 and statistically significant ($P < 0.01$). Each node represents a microbial species, and its size is proportional to the node connectivity. Each edge represents a linkage between two co-occurring nodes, and its thickness is proportional to the Spearman's correlation coefficients. All nodes are labeled with annotated species or unknown species. The shared 27 nodes between *Galaxea* and *Montipora* networks are painted using gray and the other nodes are colored at the phylum level.

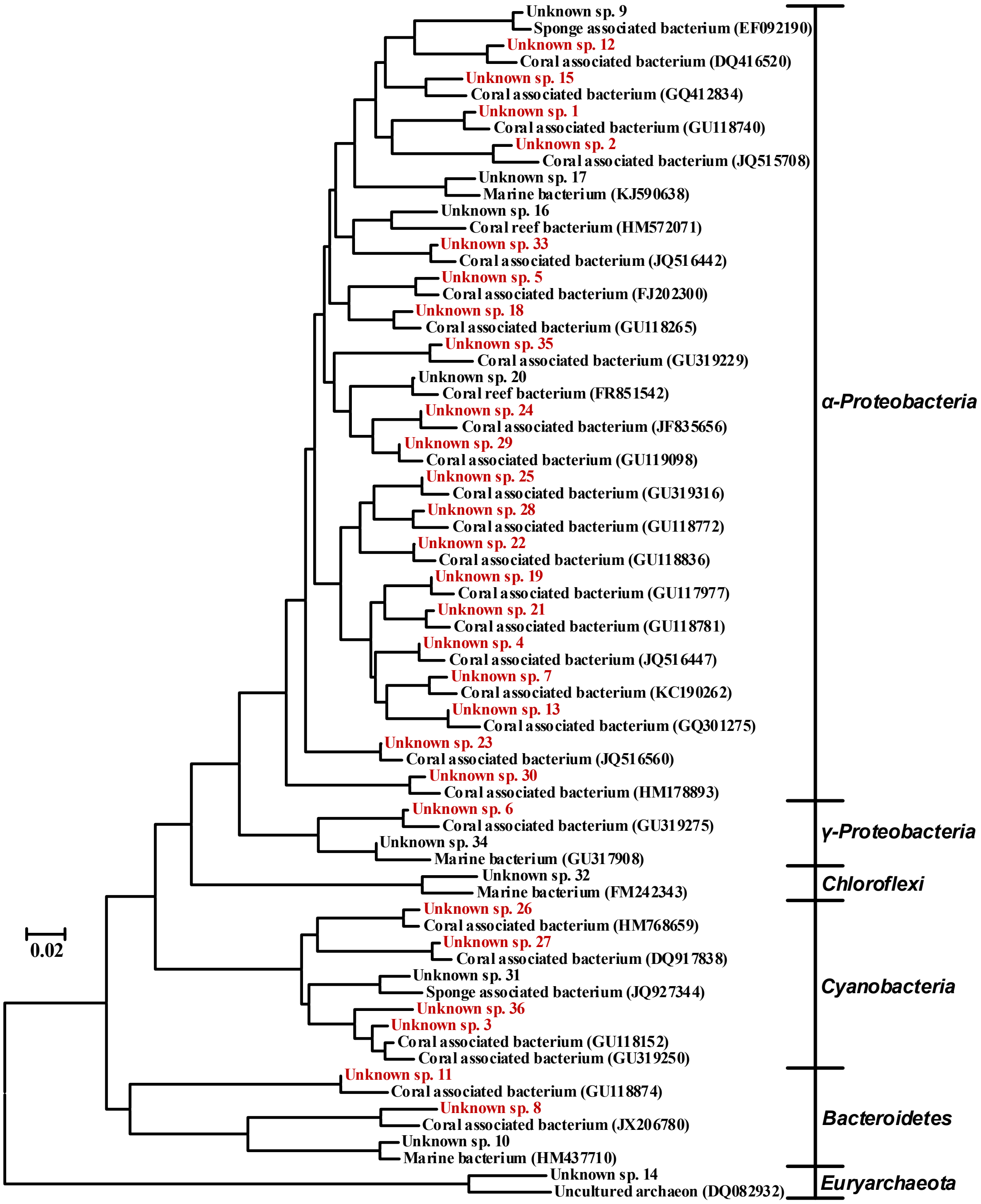

Figure S4 Phylogenetic analysis for 36 unknown co-occurring microbial species derived from *Galaxea* and *Montipra* microbiomes. MEGA 6.06 was used to construct the neighbor-joining tree tested with 1000 bootstrap replications. The scale bar 0.02 showed 2% nucleotide substitution. Reference sequences were retrieved through BLAST against NCBI nucleotide database. A total of 27 unknown species closely related to reported coral associated microbes were marked with dark red.

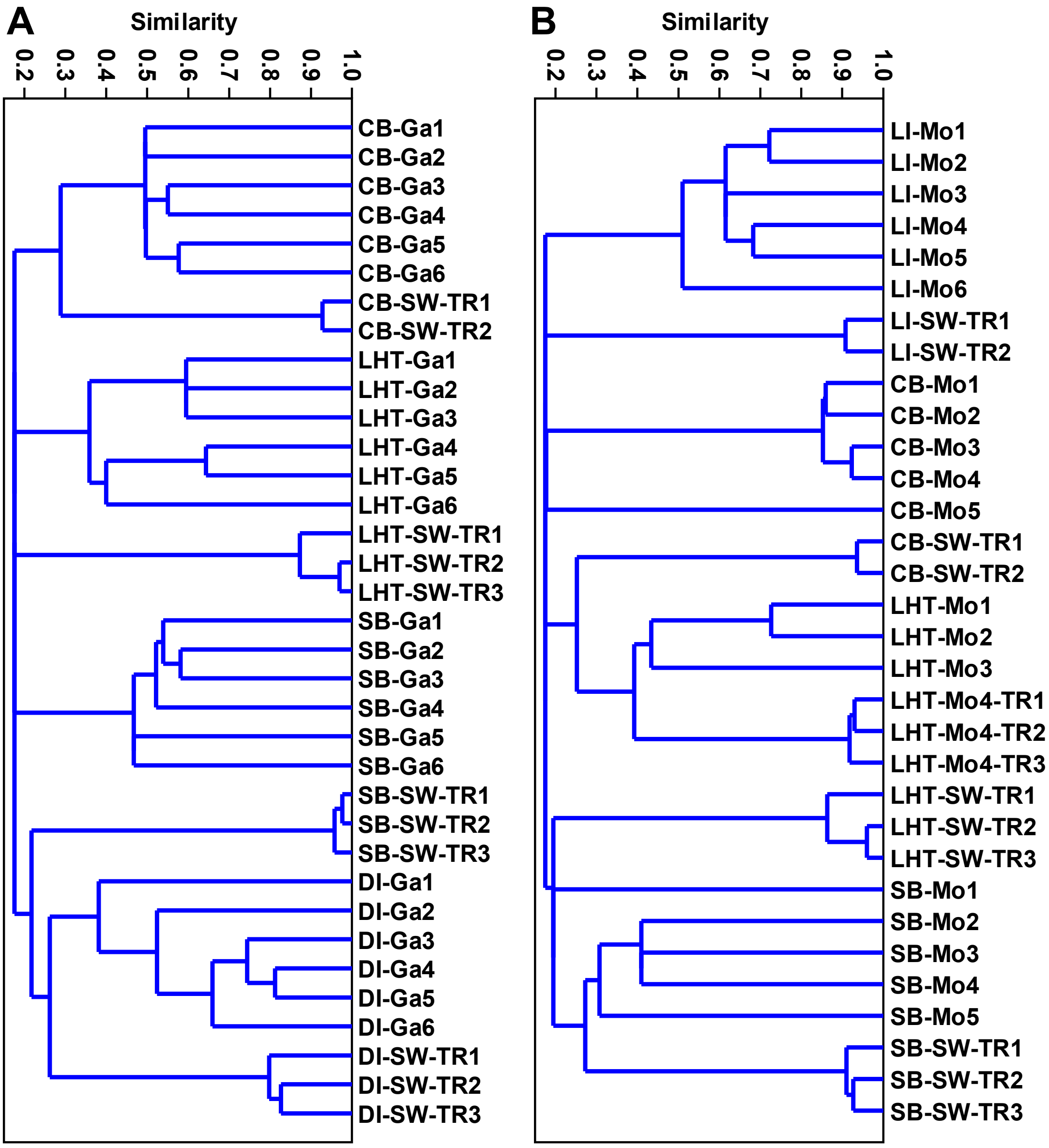

Figure S5 Cluster analysis for co-occurring microbial species derived from *Galaxea* microbiome (A) and *Montipra* microbiome (B).

Table S1 Identifications, number of clean 16S tags, number of species, and Shannon index summarized for each sample

Number	Sample IDs	Number of clean 16S tags	Number of species	Shannon index
1	CB-Ga1	5558	1688	8.9
2	CB-Ga2	6239	1234	7.8
3	CB-Ga3	2646	1093	9.2
4	CB-Ga4	5366	1540	9.2
5	CB-Ga5	3311	843	7.9
6	CB-Ga6	7348	2263	9.6
7	LHT-Ga1	3292	680	6.2
8	LHT-Ga2	1886	528	6.2
9	LHT-Ga3	2131	437	5.9
10	LHT-Ga4	6211	1758	8.7
11	LHT-Ga5	3828	1161	8.3
12	LHT-Ga6	709	329	6.8
13	SB-Ga1	981	393	6.5
14	SB-Ga2	6730	1350	7.5
15	SB-Ga3	3317	769	6.8
16	SB-Ga4	6284	1271	7.9
17	SB-Ga5	6425	914	6.6
18	SB-Ga6	1815	566	7.2
19	DI-Ga1	2422	410	4.9
20	DI-Ga2	3851	851	6.8
21	DI-Ga3	6476	822	4.9
22	DI-Ga4	2831	372	3.7
23	DI-Ga5	8463	887	4.2
24	DI-Ga6	3433	514	4.6
25	LI-Mo1	11057	2394	8.6
26	LI-Mo2	7158	1596	7.7
27	LI-Mo3	10580	1929	7.9
28	LI-Mo4	10042	2046	8.5
29	LI-Mo5	18153	2497	7.6
30	LI-Mo6	17534	2032	7.6
31	CB-Mo1	18288	892	2.8
32	CB-Mo2	22939	1365	3.5
33	CB-Mo3	29033	1218	2.9
34	CB-Mo4	23329	996	2.6
35	CB-Mo5	1743	644	8.3
36	LHT-Mo1	4638	1857	9.8
37	LHT-Mo2	9493	2585	9.5
38	LHT-Mo3	12154	1189	6.0
39	LHT-Mo4-TR1	10405	2222	7.9
40	LHT-Mo4-TR2	8436	1913	7.9

41	LHT-Mo4-TR3	7721	1761	7.8
42	SB-Mo1	3993	580	6.9
43	SB-Mo2	14438	1883	8.5
44	SB-Mo3	11721	1865	7.9
45	SB-Mo4	19684	2659	8.6
46	SB-Mo5	2586	493	5.1
47	LI-SW-TR1	9189	1322	7.2
48	LI-SW-TR2	11000	1476	7.3
49	CB-SW-TR1	14899	1346	6.4
50	CB-SW-TR2	16187	1316	6.3
51	LHT-SW-TR1	17094	2000	5.4
52	LHT-SW-TR2	13048	1383	4.6
53	LHT-SW-TR3	18609	1826	4.7
54	SB-SW-TR1	16146	1552	6.3
55	SB-SW-TR2	21273	1977	6.5
56	SB-SW-TR3	22397	2028	6.4
57	DI-SW-TR1	20574	2123	6.1
58	DI-SW-TR2	11580	1826	7.0
59	DI-SW-TR3	18751	2405	6.7

Remarks:

1. Abbreviations of sampling spots and coral species are listed in Table 1.
2. “SW” and “TR” indicated seawater and technical replicate, respectively.

Table S2 *P* and *F* value of one-way PERMANOVA for pairwise comparisons between species, locations, and seawater

P values	LHT-Ga	SB-Ga	DI-Ga	LI-Mo	CB-Mo	LHT-Mo	SB-Mo	LI-SW	CB-SW	LHT-SW	SB-SW	DI-SW
CB-Ga	0.0053	0.0022	0.0028	0.0024	0.0028	0.0020	0.0025	0.0374	0.0338	0.0127	0.0134	0.0114
LHT-Ga		0.0038	0.0025	0.0016	0.0020	0.0021	0.0040	0.0352	0.0363	0.0130	0.0128	0.0126
SB-Ga			0.0018	0.0033	0.0023	0.0020	0.0016	0.0338	0.0338	0.0118	0.0105	0.0124
DI-Ga				0.0024	0.0331	0.0025	0.0026	0.0329	0.0329	0.0110	0.0139	0.0107
LI-Mo					0.0021	0.0024	0.0025	0.0392	0.0387	0.0138	0.0105	0.0112
CB-Mo						0.0026	0.0074	0.0467	0.0483	0.0165	0.0190	0.0182
LHT-Mo							0.0035	0.0373	0.0360	0.0129	0.0096	0.0099
SB-Mo								0.0470	0.0435	0.0181	0.0192	0.0212
LI-SW									0.3337	0.1042	0.1000	0.0996
CB-SW										0.1028	0.0957	0.1021
LHT-SW											0.0994	0.0981
SB-SW												0.0976
F values	LHT-Ga	SB-Ga	DI-Ga	LI-Mo	CB-Mo	LHT-Mo	SB-Mo	LI-SW	CB-SW	LHT-SW	SB-SW	DI-SW
CB-Ga	2.706	4.050	4.946	5.368	5.580	3.720	2.112	3.911	4.346	6.513	7.562	6.465
LHT-Ga		3.241	3.971	7.629	5.446	4.558	2.693	4.531	4.900	6.276	7.714	6.568
SB-Ga			6.619	10.10	8.567	7.869	4.114	6.778	7.214	10.10	11.24	9.865
DI-Ga				11.41	1.956	7.726	4.040	5.956	6.399	7.917	9.946	8.535
LI-Mo					12.71	9.113	5.287	13.56	14.38	20.30	22.44	19.67
CB-Mo						7.447	3.985	6.709	7.149	8.872	11.07	9.718
LHT-Mo							2.692	6.729	7.220	9.933	11.51	9.811
SB-Mo								3.106	3.326	4.608	5.299	4.459
LI-SW									51.96	79.12	98.97	56.60

CB-SW										91.27	169.7	52.91
LHT-SW											119.6	62.79
SB-SW												84.24

Remark: *P* value lower than 0.05 indicates statistical significance.

Table S3 Information of 114 co-occurring microbial species involved in coral microbiome assemblages

Representative 97% OTU ID	Most similar taxonomy blasted against NCBI 16S rRNA sequence database	Identity	Final 97% OTU ID used in this study	Most similar sequence blasted against NCBI nucleotide database	Identity	Whether related to reported coral associated microbes
denovo18672	*Acinetobacter johnsonii*	98.5%	*Acinetobacter* sp. 1	N/A	N/A	Yes, very common found in corals. (Garren *et al.* 2008, Koren and Rosenberg 2008, Chen *et al.* 2011, Chiu *et al.* 2012, Lee *et al.* 2012, Morrow *et al.* 2012, Carlos *et al.* 2013, Sweet *et al.* 2013, Li *et al.* 2014, Morrow *et al.* 2015)
denovo25572	*Acinetobacter johnsonii*	98.7%	*Acinetobacter* sp. 2			
denovo28356	*Acinetobacter johnsonii*	97.6%	*Acinetobacter* sp. 3			
denovo38395	*Acinetobacter johnsonii*	99.1%	*Acinetobacter* sp. 4			
denovo5449	*Acinetobacter johnsonii*	98.0%	*Acinetobacter* sp. 5			
denovo55198	*Acinetobacter johnsonii*	97.8%	*Acinetobacter* sp. 6			
denovo23488	*Acinetobacter johnsonii*	98.1%	*Acinetobacter* sp. 7			
denovo27259	*Acinetobacter johnsonii*	98.6%	*Acinetobacter* sp. 8			
denovo34791	*Acinetobacter johnsonii*	98.3%	*Acinetobacter* sp. 9			
denovo35155	*Acinetobacter johnsonii*	97.2%	*Acinetobacter* sp. 10			
denovo36552	*Acinetobacter johnsonii*	97.4%	*Acinetobacter* sp. 11			
denovo38440	*Acinetobacter johnsonii*	97.9%	*Acinetobacter* sp. 12			
denovo4035	*Acinetobacter johnsonii*	98.8%	*Acinetobacter* sp. 13			
denovo4292	*Acinetobacter guillouiae*	99.1%	*Acinetobacter* sp. 14			
denovo44293	*Acinetobacter johnsonii*	99.2%	*Acinetobacter* sp. 15			
denovo54636	*Acinetobacter johnsonii*	98.4%	*Acinetobacter* sp. 16			
denovo9123	*Aquamicrobium ahrensii*	95.9%	*Aquamicrobium* sp.	FJ202551	99.1%	Yes (Sunagawa *et al.* 2009)
denovo30640	*Aquimarina gracilis*	98.9%	*Aquimarina* sp. 1	HM178208	97.8%	Yes (Webster *et al.* 2011)
denovo41053	*Aquimarina muelleri*	94.0%	*Aquimarina* sp. 2	JQ042974	93.6%	Sponge associated bacterium
denovo47048	*Brevundimonas vesicularis*	99.1%	*Brevundimonas* sp. 1	N/A	N/A	Yes (Cardenas *et al.* 2012)
denovo51562	*Brevundimonas naejangsanensis*	98.6%	*Brevundimonas* sp. 2			

denovo49205	*Brucella ceti*	98.9%	*Brucella* sp.	KJ611662	98.9%	Aquatic bacterium
denovo18250	*Coxiella burnetii*	96.1%	*Coxiella* sp.	EF092201	96.3%	Sponge associated bacterium
denovo53419	*Delftia acidovorans*	99.6%	*Delftia* sp.	FJ358852	99.6%	Coral reef bacterium
denovo28877	*Dichotomicrobium thermohalophilum*	94.5%	*Dichotomicrobium* sp.	JF835680	98.8%	Yes (Lins-de-Barros *et al.* 2013)
denovo37259	*Endozoicomonas euniceicola*	97.4%	*Endozoicomonas* sp. 1	N/A	N/A	Yes, very common found in corals. (Sweet *et al.* 2011, Lee *et al.* 2012, Morrow *et al.* 2012, Sweet and Bythell 2012, Bayer *et al.* 2013, Bourne *et al.* 2013, Carlos *et al.* 2013, Rodriguez-Lanetty *et al.* 2013, Lema *et al.* 2014a, Lema *et al.* 2014b, Meyer *et al.* 2014, Ainsworth *et al.* 2015, Morrow *et al.* 2015, Pantos *et al.* 2015)
denovo37449	*Endozoicomonas euniceicola*	96.5%	*Endozoicomonas* sp. 2			
denovo18975	*Endozoicomonas montiporae*	96.9%	*Endozoicomonas* sp. 3			
denovo23438	*Endozoicomonas elysicola*	93.7%	*Endozoicomonas* sp. 4			
denovo37250	*Endozoicomonas euniceicola*	96.6%	*Endozoicomonas* sp. 5			
denovo44439	*Endozoicomonas euniceicola*	95.4%	*Endozoicomonas* sp. 6			
denovo55713	*Endozoicomonas montiporae*	95.9%	*Endozoicomonas* sp. 7			
denovo36440	*Endozoicomonas montiporae*	94.0%	*Endozoicomonas* sp. 8			
denovo12081	*Endozoicomonas elysicola*	94.5%	*Endozoicomonas* sp. 9			
denovo28514	*Endozoicomonas montiporae*	93.8%	*Endozoicomonas* sp. 10			
denovo53443	*Escherichia fergusonii*	96.9%	*Escherichia* sp.	N/A	N/A	Yes (Kvennefors *et al.* 2012)
denovo44333	*Filomicrobium insigne*	96.1%	*Filomicrobium* sp.	GU319318	98.4%	Yes (Meron *et al.* 2011)
denovo2155	*Fodinicurvata sediminis*	94.0%	*Fodinicurvata* sp.	AY654833	99.1%	Yes (Unpublished)
denovo45130	*Fulvivirga imtechensis*	93.8%	*Fulvivirga* sp.	JQ516328	96.5%	Yes (Kimes *et al.* 2013)
denovo814	*Halomicronema excentricum*	94.3%	*Halomicronema* sp.	GU319174	98.9%	Yes (Meron *et al.* 2011)
denovo42096	*Hoeflea halophila*	98.2%	*Hoeflea* sp.	HM177630	99.8%	Yes (Webster *et al.* 2011)
denovo43686	*Hyphomonas adhaerens*	97.7%	*Hyphomonas* sp.	JQ515077	98.6%	Yes (Kimes *et al.* 2013)
denovo1558	*Loktanella litorea*	97.3%	*Loktanella* sp.	HE981699	98.9%	Marine bacterium
denovo37079	*Mastigocoleus testarum*	99.0%	*Mastigocoleus* sp.	JQ236254	97.8%	Yes (Meron *et al.* 2012)
denovo48281	*Methyloceanibacter caenitepidi*	97.5%	*Methyloceanibacter* sp.	EF414179	98.4%	Sponge associated bacterium
denovo52314	*Methylophilus quaylei*	98.5%	*Methylophilus* sp.	GU362946	99.3%	Marine bacterium

denovo33734	*Nitratireductor aquimarinus*	94.0%	*Nitratireductor* sp.	KC668906	99.1%	Yes (Bayer *et al.* 2013)
denovo12645	*Nitrosopumilus koreensis*	97.9%	*Nitrosopumilus* sp. 1	N/A	N/A	Yes (Sato *et al.* 2013)
denovo48748	*Nitrosopumilus maritimus*	98.3%	*Nitrosopumilus* sp. 2			
denovo32671	*Nitrospira japonica*	89.6%	*Nitrospira* sp.	N/A	N/A	Yes (Kimes *et al.* 2013)
denovo46551	*Oceanibacterium hippocampi*	94.2%	*Oceanibacterium* sp.	HM593580	99.1%	Sponge associated bacterium
denovo45562	*Pelagibacterium luteolum*	93.5%	*Pelagibacterium* sp.	GU319297	98.8%	Yes (Meron *et al.* 2011)
denovo5995	*Pelagibius litoralis*	96.5%	*Pelagibius* sp. 1	JF835655	98.6%	Yes (Lins-de-Barros *et al.* 2013)
denovo14805	*Pelagibius litoralis*	96.6%	*Pelagibius* sp. 2	JF835639	96.5%	
denovo6995	*Pelagibius litoralis*	98.8%	*Pelagibius* sp. 3	JF835639	98.8%	
denovo50691	*Phaeobacter caeruleus*	97.2%	*Phaeobacter* sp.	N/A	N/A	Yes (Bayer *et al.* 2013)
denovo13810	*Phyllobacterium myrsinacearum*	96.9%	*Phyllobacterium* sp. 1	FJ202647	99.5%	Yes (Sunagawa *et al.* 2009)
denovo50947	*Phyllobacterium myrsinacearum*	99.1%	*Phyllobacterium* sp. 2	KJ601383	99.1%	Yes (Unpublished)
denovo50174	*Prochlorococcus marinus*	96.1%	*Prochlorococcus* sp.	KJ601245	98.9%	Yes (Unpublished)
denovo27642	*Propionigenium maris*	96.6%	*Propionigenium* sp.	HM437380	97.3%	Marine bacterium
denovo7770	*Pseudahrensia aquimaris*	97.7%	*Pseudahrensia* sp.	FJ930415	99.3%	Yes (Unpublished)
denovo28681	*Pseudoruegeria aquimaris*	97.7%	*Pseudoruegeria* sp.	JN694869	98.8%	Yes (Sharp *et al.* 2012)
denovo30563	*Puniceispirillum marinum*	93.5%	*Puniceispirillum* sp.	GU981834	96.5%	Sponge associated bacterium
denovo18426	*Roseibium hamelinense*	98.6%	*Roseibium* sp.	KJ802329	99.3%	Coral reef bacterium
denovo50977	*Roseovarius aestuarii*	99.5%	*Roseovarius* sp.	N/A	N/A	Yes (Chiu *et al.* 2012)
denovo44061	*Shewanella chilikensis*	96.9%	*Shewanella* sp.	N/A	N/A	Yes (Lee *et al.* 2012)
denovo31782	*Shimia isoporae*	98.1%	*Shimia* sp.	N/A	N/A	Yes (Chiu *et al.* 2012)
denovo7878	*Sphingomonas kyungheensis*	98.4%	*Sphingomonas* sp.	KC873160	98.6%	Marine bacterium
denovo4024	*Stanieria cyanosphaera*	94.3%	*Stanieria* sp.	FJ809378	96.4%	Yes (Raina *et al.* 2009)
denovo3697	*Stenotrophomonas pavanii*	99.1%	*Stenotrophomonas* sp.	N/A	N/A	Yes (Lee *et al.* 2012)
denovo10849	*Tateyamaria omphalii*	97.7%	*Tateyamaria* sp.	JQ179398	97.7%	Yes (Webster *et al.* 2013)

denovo28119	*Tepidamorphus gemmatus*	95.6%	*Tepidamorphus* sp.	FJ202923	96.5%	Yes (Sunagawa *et al.* 2009)
denovo6468	*Thalassomonas loyana*	98.3%	*Thalassomonas* sp.	JQ178440	99.1%	Yes (Webster *et al.* 2013)
denovo29114	*Thioalkalivibrio sulfidophilus*	93.7%	*Thioalkalivibrio* sp.	FJ930590	98.7%	Yes (Unpublished)
denovo503	*Thioprofundum lithotrophicum*	95.4%	*Thioprofundum* sp. 1	AY600949	98.9%	Marine bacterium
denovo12957	*Thioprofundum lithotrophicum*	95.2%	*Thioprofundum* sp. 2	JQ727177	99.3%	Coral reef bacterium
denovo4122	*Tropicimonas isoalkanivorans*	97.2%	*Tropicimonas* sp.	FJ809237	99.1%	Yes (Raina *et al.* 2009)
denovo40314	*Vibrio pectenicida*	98.0%	*Vibrio* sp.	N/A	N/A	Yes (Chiu *et al.* 2012)
denovo18327	*Pelagibacterium luteolum*	91.9%	Unknown sp. 1	GU118740	98.2%	Yes (Sunagawa *et al.* 2010)
denovo29086	*Mesorhizobium thiogangeticum*	90.1%	Unknown sp. 2	JQ515708	96.3%	Yes (Kimes *et al.* 2013)
denovo34107	*Halomicronema excentricum*	93.2%	Unknown sp. 3	GU118152	97.7%	Yes (Sunagawa *et al.* 2010)
denovo35007	*Inquilinus ginsengisoli*	92.4%	Unknown sp. 4	JQ516447	98.8%	Yes (Kimes *et al.* 2013)
denovo36477	*Pelagibius litoralis*	93.3%	Unknown sp. 5	FJ202300	98.6%	Yes (Sunagawa *et al.* 2009)
denovo39745	*Thiohalobacter thiocyanaticus*	93.3%	Unknown sp. 6	GU319275	97.9%	Yes (Meron *et al.* 2011)
denovo4271	*Inquilinus ginsengisoli*	91.7%	Unknown sp. 7	KC190262	98.4%	Yes (Sweet *et al.* 2013)
denovo12068	*Mesonia algae*	93.1%	Unknown sp. 8	JX206780	99.1%	Sponge associated bacterium
denovo14643	*Litorimonas taeanensis*	90.1%	Unknown sp. 9	EF092190	99.3%	Sponge associated bacterium
denovo19829	*Mesonia algae*	87.7%	Unknown sp. 10	HM437710	97.8%	Marine bacterium
denovo25663	*Amoebophilus asiaticus*	87.7%	Unknown sp. 11	GU118874	98.9%	Yes (Sunagawa *et al.* 2010)
denovo30510	*Sneathiella glossodoripedis*	92.9%	Unknown sp. 12	DQ416520	97.7%	Yes (Koren and Rosenberg 2006)
denovo34715	*Azospirillum fermentarium*	90.6%	Unknown sp. 13	GQ301275	98.6%	Yes (Littman *et al.* 2009)
denovo53087	*Uncultured archaeon clone*	92.8%	Unknown sp. 14	DQ082932	92.8%	No close relative
denovo53090	*Dichotomicrobium thermohalophilum*	92.3%	Unknown sp. 15	GQ412834	96.8%	Yes (Garren *et al.* 2009)
denovo56	*Parvibaculum hydrocarboniclasticum*	92.3%	Unknown sp. 16	HM572071	93.6%	Coral reef bacterium
denovo7091	*Anderseniella baltica*	91.2%	Unknown sp. 17	KJ590638	97.5%	Marine bacterium
denovo11833	*Limimonas halophila*	93.2%	Unknown sp. 18	GU118265	97.5%	Yes (Sunagawa *et al.* 2010)

denovo12057	*Nisaea denitrificans*	92.0%	Unknown sp. 19	GU117977	99.3%	Yes (Sunagawa *et al.* 2010)
denovo14499	*Pelagibius litoralis*	92.1%	Unknown sp. 20	FR851542	99.1%	Coral reef bacterium
denovo22408	*Tistlia consotensis*	91.5%	Unknown sp. 21	GU118781	98.8%	Yes (Sunagawa *et al.* 2010)
denovo23302	*Tistlia consotensis*	92.2%	Unknown sp. 22	GU118836	99.3%	Yes (Sunagawa *et al.* 2010)
denovo27834	*Oceanibaculum pacificum*	92.2%	Unknown sp. 23	JQ516560	99.1%	Yes (Kimes *et al.* 2013)
denovo28013	*Pelagibius litoralis*	92.6%	Unknown sp. 24	JF835656	98.8%	Yes (Lins-de-Barros *et al.* 2013)
denovo30174	*Oceanibaculum pacificum*	93.2%	Unknown sp. 25	GU319316	99.5%	Yes (Meron *et al.* 2011)
denovo34667	*Halospirulina tapeticola*	92.9%	Unknown sp. 26	HM768659	99.1%	Yes (Klaus *et al.* 2011)
denovo3521	*Loriellopsis cavernicola*	92.1%	Unknown sp. 27	DQ917838	98.4%	Yes (Unpublished)
denovo43089	*Oceanibaculum pacificum*	91.6%	Unknown sp. 28	GU118772	97.9%	Yes (Sunagawa *et al.* 2010)
denovo43192	*Tistlia consotensis*	92.8%	Unknown sp. 29	GU119098	99.5%	Yes (Sunagawa *et al.* 2010)
denovo46771	*Geminicoccus roseus*	91.0%	Unknown sp. 30	HM178893	97.5%	Yes (Webster *et al.* 2011)
denovo49470	*Halomicronema excentricum*	91.0%	Unknown sp. 31	JQ927344	96.8%	Sponge associated bacterium
denovo52824	*Caldilinea tarbellica*	84.5%	Unknown sp. 32	FM242343	95.1%	Marine bacterium
denovo52933	*Tepidamorphus gemmatus*	92.1%	Unknown sp. 33	JQ516442	98.6%	Yes (Kimes *et al.* 2013)
denovo55984	*Spongiibacter tropicus*	92.6%	Unknown sp. 34	GU317908	97.4%	Marine bacterium
denovo56622	*Azospirillum fermentarium*	90.8%	Unknown sp. 35	GU319229	97.5%	Yes (Meron *et al.* 2011)
denovo5836	*Halomicronema excentricum*	92.9%	Unknown sp. 36	GU319250	95.4%	Yes (Meron *et al.* 2011)

Remarks:

1. The species *Acinetobacter* spp., *Brevundimonas* spp., *Endozoicomonas* spp., *Escherichia* sp., *Nitrosopumilus* spp., *Nitrospira* sp., *Phaeobacter* sp., *Roseovarius* sp., *Shewanella* sp., *Shimia* sp., *Stenotrophomonas* sp., and *Vibrio* sp. were reported as coral associated microbes. The other species including those unknown species with 16S identity lower than 94% to NCBI microbial taxonomy were blasted again against NCBI nucleotide database to find out whether or not they were closely related to coral associated microbes.
2. "N/A" indicated not applicable.

References for Table S3

Ainsworth TD, Krause L, Bridge T, Torda G, Raina JB, Zakrzewski M *et al.* (2015). The coral core microbiome identifies rare bacterial taxa as ubiquitous endosymbionts. *ISME J***:** Epub ahead of print.

Bayer T, Neave MJ, Alsheikh-Hussain A, Aranda M, Yum LK, Mincer T *et al.* (2013). The microbiome of the Red Sea coral *Stylophora pistillata* is dominated by tissue-associated *Endozoicomonas* Bacteria. *Appl Environ Microbiol* **79:** 4759-4762.

Bourne DG, Dennis PG, Uthicke S, Soo RM, Tyson GW, Webster N. (2013). Coral reef invertebrate microbiomes correlate with the presence of photosymbionts. *ISME J* **7:** 1452-1458.

Cardenas A, Rodriguez RL, Pizarro V, Cadavid LF, Arevalo-Ferro C. (2012). Shifts in bacterial communities of two Caribbean reef-building coral species affected by white plague disease. *ISME J* **6:** 502-512.

Carlos C, Torres TT, Ottoboni LM. (2013). Bacterial communities and species-specific associations with the mucus of Brazilian coral species. *Sci Rep* **3:** 1624.

Chen CP, Tseng CH, Chen CA, Tang SL. (2011). The dynamics of microbial partnerships in the coral *Isopora palifera*. *ISME J* **5:** 728-740.

Chiu JMY, Li S, Li A, Po B, Zhang R, Shin PKS *et al.* (2012). Bacteria associated with skeletal tissue growth anomalies in the coral *Platygyra carnosus*. *FEMS Microbiol Ecol* **79:** 380-391.

Garren M, Smriga S, Azam F. (2008). Gradients of coastal fish farm effluents and their effect on coral reef microbes. *Environ Microbiol* **10:** 2299-2312.

Garren M, Raymundo L, Guest J, Harvell CD, Azam F. (2009). Resilience of coral-associated bacterial communities exposed to fish farm effluent. *PLoS One* **4:** e7319.

Kimes NE, Johnson WR, Torralba M, Nelson KE, Weil E, Morris PJ. (2013). The *Montastraea faveolata* microbiome: ecological and temporal influences on a Caribbean reef-building coral in decline. *Environ Microbiol* **15:** 2082-2094.

Klaus JS, Janse I, Fouke BW. (2011). Coral black band disease microbial communities and genotypic variability of the dominant cyanobacteria (Cd1c11). *B Mar Sci* **87:** 795-821.

Koren O, Rosenberg E. (2006). Bacteria associated with mucus and tissues of the coral *Oculina patagonica* in summer and winter. *Appl Environ Microbiol* **72:** 5254-5259.

Koren O, Rosenberg E. (2008). Bacteria associated with the bleached and cave coral *Oculina patagonica*. *Microb Ecol* **55:** 523-529.

Kvennefors ECE, Sampayo E, Kerr C, Vieira G, Roff G, Barnes AC. (2012). Regulation of bacterial communities through antimicrobial activity by the coral holobiont. *Microb Ecol* **63:** 605-618.

Lee OO, Yang JK, Bougouffa S, Wang Y, Batang Z, Tian RM *et al.* (2012). Spatial and species variations in bacterial communities associated with corals from the Red Sea as revealed by pyrosequencing. *Appl Environ Microbiol* **78:** 7173-7184.

Lema KA, Bourne DG, Willis BL. (2014a). Onset and establishment of diazotrophs and other bacterial associates in the early life history stages of the coral *Acropora millepora*. *Mol Ecol* **23:** 4682-4695.

Lema KA, Willis BL, Bourne DG. (2014b). Amplicon pyrosequencing reveals spatial and temporal consistency in diazotroph assemblages of the *Acropora millepora* microbiome. *Environ Microbiol* **16:** 3345-3359.

Li J, Chen Q, Long LJ, Dong JD, Yang J, Zhang S. (2014). Bacterial dynamics within the mucus, tissue and skeleton of the coral *Porites lutea* during different seasons. *Sci Rep* **4:** 7320.

Lins-de-Barros MM, Cardoso AM, Silveira CB, Lima JL, Clementino MM, Martins OB *et al.* (2013). Microbial community compositional shifts in bleached colonies of the Brazilian reef-building coral *Siderastrea stellata*. *Microb Ecol* **65:** 205-213.

Littman RA, Willis BL, Bourne DG. (2009). Bacterial communities of juvenile corals infected with different *Symbiodinium* (dinoflagellate) clades. *Mar Ecol Prog Ser* **389:** 45-59.

Meron D, Atias E, Iasur Kruh L, Elifantz H, Minz D, Fine M *et al.* (2011). The impact of reduced pH on the microbial community of the coral *Acropora eurystoma*. *ISME J* **5:** 51-60.

Meron D, Rodolfo-Metalpa R, Cunning R, Baker AC, Fine M, Banin E. (2012). Changes in coral microbial communities in response to a natural pH gradient. *ISME J* **6:** 1775-1785.

Meyer JL, Paul VJ, Teplitski M. (2014). Community shifts in the surface microbiomes of the coral *Porites astreoides* with unusual lesions. *PLoS One* **9:** e100316.

Morrow KM, Moss AG, Chadwick NE, Liles MR. (2012). Bacterial associates of two Caribbean coral species reveal species-specific distribution and geographic variability. *Appl Environ Microbiol* **78:** 6438-6449.

Morrow KM, Bourne DG, Humphrey C, Botte ES, Laffy P, Zaneveld J *et al.* (2015). Natural volcanic CO_2 seeps reveal future trajectories for host-microbial associations in corals and sponges. *ISME J* **9:** 894-908.

Pantos O, Bongaerts P, Dennis PG, Tyson GW, Hoegh-Guldberg O. (2015). Habitat-specific environmental conditions primarily control the microbiomes of the coral *Seriatopora hystrix*. *ISME J***:** Epub ahead of print.

Raina JB, Tapiolas D, Willis BL, Bourne DG. (2009). Coral-associated bacteria and their role in the biogeochemical cycling of sulfur. *Appl Environ Microbiol* **75:** 3492-3501.

Rodriguez-Lanetty M, Granados-Cifuentes C, Barberan A, Bellantuono AJ, Bastidas C. (2013). Ecological inferences from a deep screening of the complex bacterial consortia associated with the coral, *Porites astreoides*. *Mol Ecol* **22:** 4349-4362.

Sato Y, Willis BL, Bourne DG. (2013). Pyrosequencing-based profiling of archaeal and bacterial 16S rRNA genes identifies a novel archaeon associated with black band disease in corals. *Environ Microbiol* **15:** 2994-3007.

Sharp KH, Distel D, Paul VJ. (2012). Diversity and dynamics of bacterial communities in early life stages of the Caribbean coral *Porites astreoides*. *ISME J* **6:** 790-801.

Sunagawa S, DeSantis TZ, Piceno YM, Brodie EL, DeSalvo MK, Voolstra CR *et al.* (2009). Bacterial diversity and White Plague Disease-associated community changes in the Caribbean coral *Montastraea faveolata*. *ISME J* **3:** 512-521.

Sunagawa S, Woodley CM, Medina M. (2010). Threatened corals provide underexplored microbial habitats. *PLoS One* **5:** e9554.

Sweet M, Bythell J. (2012). Ciliate and bacterial communities associated with White Syndrome and Brown Band Disease in reef-building corals. *Environ Microbiol* **14:** 2184-2199.

Sweet M, Burn D, Croquer A, Leary P. (2013). Characterisation of the bacterial and fungal communities associated with different lesion sizes of dark spot syndrome occurring in the coral *Stephanocoenia intersepta*. *PLoS One* **8:** e62580.

Sweet MJ, Croquer A, Bythell JC. (2011). Dynamics of bacterial community development in the reef coral *Acropora muricata* following experimental antibiotic treatment. *Coral Reefs* **30:** 1121-1133.

Webster NS, Soo R, Cobb R, Negri AP. (2011). Elevated seawater temperature causes a microbial

shift on crustose coralline algae with implications for the recruitment of coral larvae. *ISME J* **5:** 759-770.

Webster NS, Uthicke S, Botte ES, Flores F, Negri AP. (2013). Ocean acidification reduces induction of coral settlement by crustose coralline algae. *Global Change Biol* **19:** 303-315.
